# ST proteins, a new family of plant tandem repeat proteins with a DUF2775 domain mainly found in Fabaceae and Asteraceae

**DOI:** 10.1186/1471-2229-12-207

**Published:** 2012-11-07

**Authors:** Lucía Albornos, Ignacio Martín, Rebeca Iglesias, Teresa Jiménez, Emilia Labrador, Berta Dopico

**Affiliations:** 1 Dpto. de Fisiología Vegetal, Centro Hispano Luso de Investigaciones Agrarias (CIALE), Universidad de Salamanca, Plaza Doctores de la Reina s/n. Campus Miguel Unamuno, Salamanca, 37007, Spain

**Keywords:** Biotic interaction, DUF2775 domain, ST proteins, Tandem repeat proteins

## Abstract

**Background:**

Many proteins with tandem repeats in their sequence have been described and classified according to the length of the repeats: I) Repeats of short oligopeptides (from 2 to 20 amino acids), including structural cell wall proteins and arabinogalactan proteins. II) Repeats that range in length from 20 to 40 residues, including proteins with a well-established three-dimensional structure often involved in mediating protein-protein interactions. (III) Longer repeats in the order of 100 amino acids that constitute structurally and functionally independent units. Here we analyse **S**hoo**T** specific (ST) proteins, a family of proteins with tandem repeats of unknown function that were first found in Leguminosae, and their possible similarities to other proteins with tandem repeats.

**Results:**

ST protein sequences were only found in dicotyledonous plants, limited to several plant families, mainly the Fabaceae and the Asteraceae. *ST* mRNAs accumulate mainly in the roots and under biotic interactions. Most ST proteins have one or several Domain(s) of Unknown Function 2775 (DUF2775). All deduced ST proteins have a signal peptide, indicating that these proteins enter the secretory pathway, and the mature proteins have tandem repeat oligopeptides that share a hexapeptide (E/D)FEPRP followed by 4 partially conserved amino acids, which could determine a putative N-glycosylation signal, and a fully conserved tyrosine. In a phylogenetic tree, the sequences clade according to taxonomic group. A possible involvement in symbiosis and abiotic stress as well as in plant cell elongation is suggested, although different STs could play different roles in plant development.

**Conclusions:**

We describe a new family of proteins called ST whose presence is limited to the plant kingdom, specifically to a few families of dicotyledonous plants. They present 20 to 40 amino acid tandem repeat sequences with different characteristics (signal peptide, DUF2775 domain, conservative repeat regions) from the described group of 20 to 40 amino acid tandem repeat proteins and also from known cell wall proteins with repeat sequences. Several putative roles in plant physiology can be inferred from the characteristics found.

## Background

One characteristic of many proteins is the presence of tandem repeats in their sequence. It has been estimated that approximately 14% of all known proteins contain significant internal repeats, most of them in eukaryotic organisms [1].

Different categories of tandem repeats can be defined [2]. (I) Repeats of short oligopeptides (from 2 to 20 amino acids) that are unlikely to form structural or functional units by themselves and become a single functional entity when interactions among repeats take place. (II) Repeats that range in length from 20 to 40 residues, which form structurally independent units but may not function independently [3]. Within this group there are proteins with a well-established three-dimensional structure that are often involved in mediating protein-protein interactions. (III) Longer repeats in the order of 100 amino acids that constitute structurally and functionally independent units [3].

Structural cell wall proteins as well as arabinogalactan proteins (AGPs) are included in the first category described above. There are three main classes of structural proteins in plant cell walls; the division is based on their amino acid composition and their repeating sequences: hydroxyproline-rich glycoproteins or extensins (HRGPs), proline-rich proteins (PRPs), and glycine-rich proteins (GRPs) [4,5]. HRGPs contain Ser-Hyp_4_ pentapeptide repeats, which are glycosylated by galactose and arabinose side chains [6] and whose role is to strengthen the cell wall [7]. PRPs have been classified in five classes [8] on the basis of their motifs, domains and biochemical characteristics, and they were first identified as proteins that accumulate in the cell wall in response to physical damage [9]. They have also been implicated in cell wall integrity [10] and in plant defence [11]. GRPs are characterized by a high content (more than 60%) and repetitive sequences of glycine residues based on (Gly-X)_n_ motifs. Cell wall GRPs are often associated with the vascular system of plants, and their synthesis appears to be regulated during development [12].

AGPs are proteoglycans whose carbohydrate part accounts for more than 90% of the molecule on a weight basis [13]. In many AGPs the protein core contains relatively high amounts of hydroxyproline, serine, threonine and alanine [13]. AGPs have been classified as classical AGPs, which contain a glycosylphophatidylinositol (GPI) anchor signal sequence for plasma membrane attachment [14]; non-classical AGPs, which do not contain a GPI signal, AG-peptides of 10- to 15 amino acid residues, and fasciclin-like AGPs. AGPs have been implicated in various plant growth and developmental processes (see review by Seifert and Roberts [15]), such as somatic embryogenesis, pollen tube growth, cell proliferation, cell expansion, and cell differentiation.

The second category of tandem repeat proteins, those with repeats of between 20 and 40 amino acids, are classified attending to the consensus sequence of the repeat. Some well characterized repeats are ankyrin [16], HEAT [3,17], a leucine-rich repeat (LRR) [18], armadillo [3,19], Kelch [20], WD-40 [21], tetratricopeptide repeat (TPR) [3,22], pentatricopeptide repeat (PPR) [23], α-coiled coil heptad repeats [24] and an eicosapentapeptide repeat (EPR) [25]. Most of these repeats have a significant preference for binding proteins and other ligands; this capacity is determined by the tertiary structure. Thus, WD-40 and Kelch repeats fold in ß-propellers [26,27]. These structures have a binding “supersite” along the propeller axis with a preference for small ligands. Instead TPRs form a right-handed super-helix with a groove of large surface area available for ligand binding and they do not appear to possess a single supersite. HEAT proteins form antiparallel α helices stacked in a consecutive array resembling a hook [3], and LRRs adopt a curved shape with α helices and ß strands and their repeats also participate in protein-protein or protein-ligand interactions (rev. in Andrade et al. [28]). Nevertheless, many tandem repeats seem to exhibit only a slight tendency to form a single, well-defined structure. Most cannot be crystallized; if the parent protein is crystallized, the tandem repeat region has weak electron density and appears disordered. Many repeats are, or consist of, Intrinsically Unstructured Proteins (IUPs) [29]; in turn many IUP’s consist of tandem repeats. However, there are many exceptions both ways.

Tandem repeats confer important structural features and special functions to these proteins but they usually occur in association with other domains with specific biological roles, and consequently proteins in the same family may perform dissimilar functions. As an a example, in plants many proteins with different roles have LRR domains, such as TRANSPORT INHIBITOR RESPONSE 1 (TIR1), the auxin receptor involved in the auxin signalling pathway [30]; the protein encoded by tomato Cf9 [31], which is an R protein and participates in plant disease resistance, and CLAVATA 1 (CLV1) a Ser/Thr kinase receptor, necessary for the maintenance of the shoot apical meristem [32].

The aim of the present work was to analyze **S**hoo**T** specific (ST) proteins, a class of proteins with tandem repeats from 20 to 40 amino acids. *ST* mRNAs were first found in leguminosae [33,34], but at that time no similar transcripts had been found in other species. Nevertheless, the present increase in genome sequencing projects and expressed sequence tags (EST) databases in recent years has allowed the search for new similar *ST* DNA and/or ST protein sequences. We analyzed the presence of the ST gene/protein family in living organisms with a view to finding clues about the structure and role of such proteins. We also studied their possible similarities to other proteins of tandem repeats including cell wall proteins in order to determine whether they belong to any of these groups.

The first *ST* cDNA (C3, here called *PsaST2*) was found in *Pisum sativum*[33,35]. It was described as shoot-/stem-specific, having as its main characteristics 4 tandem repeats of 26 amino acids and a putative signal peptide, as well as several putative N-glycosylation sites. In 1990, using C3 as a probe Williams et al. [36] found 2 genes in the pea genome, one of them highly expressed in stem (called S2 and equivalent to C3) and the other one expressed in pea pod tissues, but not in stem (S4, here referred to as *PsaST3*). Those authors also pointed out the existence of a third related gene. The proteins encoded by these genes were described as organ-specific and differed in the number of repeats.

Later, two ST-related clones of *Cicer arietinum CanST1* and *CanST2* (called *CarST1* and *CarST2* in this paper), from a 5-day old epicotyl cDNA library were isolated after a differential screening to select clones whose expression increases with epicotyl growth [34]. The possible existence of at least one more gene in chickpea was also noted. The chickpea CarST2 deduced protein presents the highest homology with pea sequences except that it has 8 repeats. CarST1 is slightly different, mainly because there are 25 amino acids in each of its 7 tandem repeat sequences and no putative N-glycosylation sites are present [34]. As in peas, chickpea STs are also organ-specific, and transcript accumulation is limited to organs that perform elongation in a way not related to auxin or to brassinolides [34], and appear just when the epicotyl starts to elongate after germination [37]. The role of these proteins in plant development remains unknown, although a putative role in cell elongation has been suggested [33,34,37].

## Results and discussion

### STs are specific to several plant families and form a multigenic family

**Figure 1:**
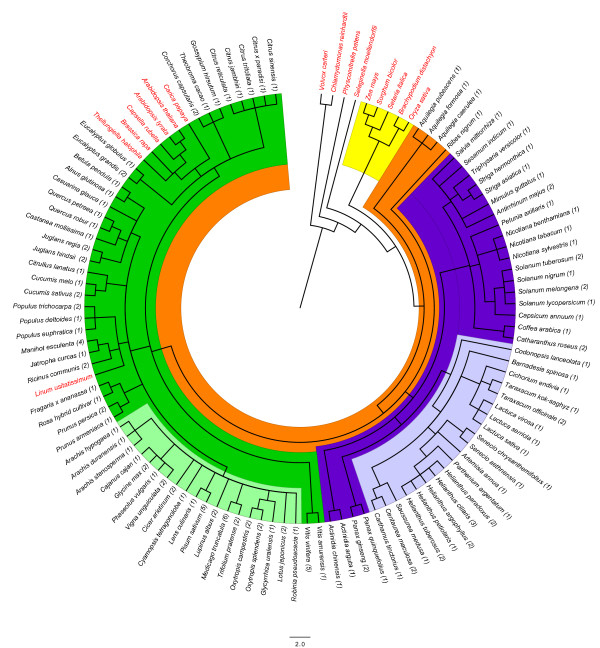
**Phylogenetic tree of species having ST sequences.** The phylogenetic tree was done with the Common Tree Taxonomy tool at NCBI and edited using FigTree v1.3.1. All species with ST proteins belong to Magnoliophyta (highlighted in orange) mainly in Asteridae (39%, highlighted in purple) and Rosidae (59%, highlighted in green). Two taxonomic families, the Fabaceae (24%, highlighted in light green) and the Asteraceae (19%, highlighted in light purple) grouped 43% of the genera found. The green algae, moss, lycophyte (no highlighted) as well as monocots (highlighted in yellow) do not have ST sequences. Numbers in brackets indicate the number of ST sequences found in each species.

All ST sequences found in the different searches conducted in this work belonged to dicot plants (taxonomic Class Magnoliopsida) and no similar sequences were found in kingdoms other than the plant kingdom. According to NCBI taxonomic classification, the phylogenetic distribution of all species having ST proteins as well as a few representative species without ST proteins (Figure 1) showed that no *ST* genes were found in green algae (*Volvox carteri* and *Chlamydomonas reinhardtii*), moss (*Physcomitrella patens*) and lycophyte (*Selaginella moellendorffii*) genomes. Also no ST-encoding sequences or ST proteins were found in gymnosperms or monocots (Figure 1). Since several monocot genomes, such as rice or maize, are now fully sequenced it may be concluded that this kind of protein is not present in these plants.

Taking into account genes, ESTs, and protein searches, sequences encoding proteins similar to ST (listed in Additional file 1) were found in 65 plant genera and 98 species (see Additional file 1) belonging to Magnoliophyta (orange in Figure 1) mainly in Asteridae (39%, purple in Figure 1) and Rosidae (59%, green in Figure 1). Two taxonomic families, the Fabaceae family (24%, light green in Figure 1) and the Asteraceae family (19%, light purple in Figure 1), grouped 43% of the genera found. It is worth noting that no sequences encoding ST proteins were found in dicot species such as the Brassicales *Arabidopsis thaliana* and *Carica papaya*, among others, and *Linum usitatissimum* whose genomes have been fully sequenced. Nonetheless, it is possible that more species, both with and without *ST* genes, could appear as the genome sequencing projects progressed. Up to date the plant species that do not appear in the phylogenetic tree (Figure 1) are not known to encode ST proteins.

After the USDA Plants Databases taxonomic classification ( http://plants.usd.gov/java/), all species are included in five subclasses among the 6 described in the class Magnoliopsida: Asteridae (7 orders, 8 families, 24 genera, 37%), Rosidae (7 orders, 8 families, 27 genera, 42%), Dilleniidae (4 orders, 6 families, 7 genera, 11%), Hamamelididae (3 orders, 4 families, 6 genera, 9%) and Magnoliidae (1 order, 1 family, 1 genus, 1%).

Among the 27 genomes found on the phytozome v7.0 web page ( http://www.phytozome.net/) there are *ST* genes in only 11 plant species (Table 1): *Manihot esculenta*, *Ricinus communis*, *Populus trichocarpa*, *Medicago truncatula*, *Glycine max*, *Cucumis sativus*, *Prunus persica*, *Eucalyptus grandis*, *Vitis vinifera*, *Mimulus guttatus* and *Aquilegia caerulea*. Although searches to find *ST* genes retrieve many low-similarity sequences in most genomes, several matches do not have an associated ORF or the peptides do not show ST characteristics, as described below.

**Table 1:** ***ST *genes found in the genomes available from the v7.0. phytozome web page**

**Number**	**Gene name**	**Locus gene or Acc number**	**Species**
328	*AcaST2*	AcoGoldSmith_v1.012977m.g	*Aquilegia caerulea*
329	CsaST1*	Cucsa.240460	*Cucumis sativus*
330	CsaST2*	Cucsa.240450	*Cucumis sativus*
331	*EgrST2*	Egrandis_v1_0.030546m.g	*Eucalyptus grandis*
340	*EgrST3*	Egrandis_v1_0.054954m.g	*Eucalyptus grandis*
341	*GmaST1*	Glyma11g12650	*Glycine max*
342	*GmaST2*	Glyma06g01540	*Glycine max*
343	*MesST2*	cassava45580.m1	*Manihot esculenta*
344	*MesST3*	cassava10690.m1	*Manihot esculenta*
345	*MesST4*	cassava4.1_023025m.g	*Manihot esculenta*
346	*MesST5*	cassava10702.m1	*Manihot esculenta*
347	*MtrST1*	Medtr4g095360	*Medicago truncatula*
348	*MtrST2*	Medtr3g159540	*Medicago truncatula*
349	*MtrST3*	Medtr3g159550 (TC 117542)	*Medicago truncatula*
350	*MtrST4*	Medtr3g041180 (TC 122142)	*Medicago truncatula*
351	*MtrST5*	Medtr3g041160	*Medicago truncatula*
352	*MtrST6*	Medtr3g136390 (TC 107197)	*Medicago truncatula*
353	*MguST2*	Mgf014516m	*Mimulus guttatus*
354	*PtrST2*	POPTR_009s11770	*Populus trichocarpa*
355	*PtrST3*	POPTR_009s11760	*Populus trichocarpa*
356	*PpeST2*	ppa010784m.g	*Prunus persica*
357	*PpeST3*	ppa013282m.g	*Prunus persica*
358	*RcoST2*	30190.m010883	*Ricinus comunis*
359	RcoST3*	29851.m002425	*Ricinus comunis*
360	*VviST2*	GSVIVT00036405001	*Vitis vinifera*
361	*VviST3*	GSVIVT00036409001	*Vitis vinifera*
362	VviST4*	GSVIVT00036410001	*Vitis vinifera*
363	VviST5*	GSVIVT00036406001	*Vitis vinifera*
364	VviST6*	GSVIVT01031943001	*Vitis vinifera*

Genes that did not encode a canonical ST as determined by studying the protein sequences are indicated by *. Some of the genes were also found in EMBL genebank, as can be seen in Additional file 1. Numbers indicate the order in which the sequences were found.

Among type-II tandem repeat proteins [2], proteins with EPR repeats have also only been described in angiosperms [25], although unlike STs they have also been found in 20 monocot species such as rice, and in 45 dicots such as arabidopsis, indicating a further specialization of ST proteins.

In light of these data, it seems clear that ST proteins are limited to several taxonomic families, which indicates that their function could be related to differential characteristics of the families in which they are present. However, the fact that they are not ubiquitous proteins in the plant kingdom does not indicate a minor role in plant development.

As initially reported for pea [33,36] and chickpea [34], analyses of the genomes of plant species having *ST* indicated that they have at least two *ST* genes (Table 1), except for *A*. *caerulea* and *M*. *guttatus*. The largest family was found in *M*. *truncatula*, with six members, followed by *V*. *vinifera*, with 5 genes, and *M*. *esculenta* with 4 (Table 1). Analysis of EST fragments also pointed to the existence of a family of *ST* genes in several species. Five different EST-encoding ST proteins were found in *P*. *sativum* and 2 in *Helianthus ciliaris*, as summarized in Additional file 2. Furthermore, it is possible that not all the members of each species have yet been isolated.

**Figure 2:**
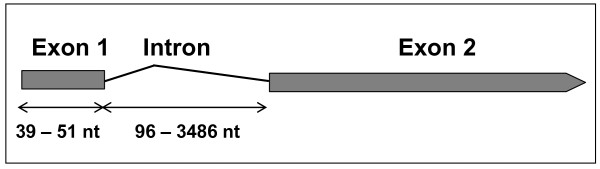
**Organization of the canonical ST gene.** The gene has one intron starting 39 to 51 nucleotides from the initial methionine codon, ranging from 96 to 3486 nucleotides in length.

Most genes studied (19 out of 29), or at least canonical *ST* genes, have one intron starting 39 to 51 nucleotides from the initial methionine codon, ranging from 96 to 3486 nucleotides in length (Figure 2). The presence of such an intron makes it difficult the characterization of full-length open reading frames (ORFs) in genome analyses, owing to the occurrence of several methionine-encoding codons downstream from the intron. Thus, in *C*. *sativus ST1* and *ST2* (*CsaST1* and *CsaST2*), *P*. *persica ST2* (*PpeST2*) and *M*. *esculenta ST4* (*MesST4*) genes no intron has been defined, and in *R*. *communis ST3* (*RcoST3*), *V*. *vinifera ST4* (*VviST4*) and *A*. *caerulea ST2* (*AcaST2*) the intron starts at nucleotides 169, 329 and 5, respectively. Finally in *VviST5*, *MesST2* and *E*. *grandis ST3* (*EgrST3*) two introns were determined. The full-length ORF in *ST* genes ranged from 403 (*RcoST2*) to 1482 nucleotides (*MtrST4*). Finally, full-length EST sequences clearly establish the presence of the intron upon comparing genomic and cDNA sequences.

### ST proteins present a signal peptide, a DUF2775 domain and tandem repeat oligopeptides and could be grouped in three main types

After the search performed here, we studied a large number of deduced ST proteins in order to look for common features. Since no purified ST protein has ever been isolated, our data come from the gene or cDNA deduced proteins and some characteristics remain to be confirmed empirically.

Since most cDNA deduced proteins came from studies of massive EST characterization, an analysis of the proteins was performed in order to determine whether each EST fragment encoded a full-length protein and the number of different ST proteins in each species. To date, 136 different ST proteins have been found (the number of different ST in each species is shown in Additional file 2 and the protein sequences can be found in Additional file 3). Analysis of these proteins revealed that 72 were full-length proteins; 38 had at least the N-terminal sequence; in 7 it seemed that the C-terminal end was complete, and finally 19 sequences represented only internal fragments of the protein.

ST proteins vary in size. After the analysis of 72 ST full-length sequences, a variation between 103 (PgiST2) and 493 (MtrST4) amino acids in length was found, and hence the predicted molecular mass varied from 12.0 to 56.0 kDa, in most cases being a characteristic of the plant species in question. Thus, the *V*. *vinifera* family ranged from 142 to 146 amino acids and the *M*. *truncatula* family from 359 to 493. Thirty-six percent of the ST sequences had fewer than 150 amino acids; 41% ranged from 150 to 250, and 23% had more than 250 amino acids. This latter group included the members of the barrel medic ST family, 5 ST proteins of the Fabaceae family, including chickpea, 3 sequences of Asteraceae belonging to the genus *Helianthus* as well as JcuST2, MesST3 and StuST2. The predominance of smaller ST proteins could have been due to the way in which the sequences were found; analysis of EST fragments usually gives the smallest one in higher proportions. Thus, analysis of the sequences that were not full-length showed that 62% had more than 150 amino acids, of which 56% had more than 200 amino acids.

**Figure 3:**
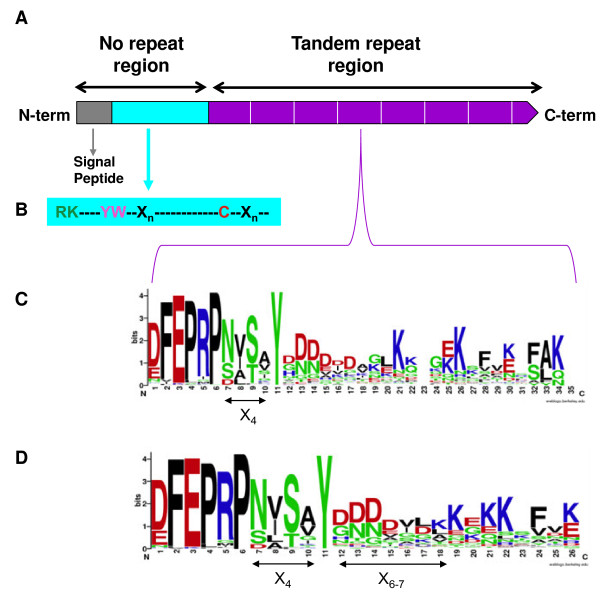
**Organization of the ST proteins.** (**A**) The signal peptide, the mature N-terminal end before repeats and the tandem repeat region are shown. (**B**) Detail of amino acid sequence of the N-terminal end of the mature protein. (**C, D**) Details of the tandem repeat oligopeptides represented using WebLogo. (**C**) Comparison of ST protein repeats having different sizes, showing the conserved consensus sequence DFEPRPX_4_Y. (**D**) Comparison of the most abundant ST protein repeats having 25 and 26 amino acids. The general consensus sequence for each repeat is DFEPRPX_4_YX_6-7_KXKKXFXK, which shows a less conserved K at positions 19, 21, 22 and 26 (26 amino acids repeats) or 18, 20, 21 and 25 (25 amino acids repeats). Apart from X_4_ positions, amino acids X_6-7_ in the consensus pattern are phylogenetically conserved, being rich in D and N. The WebLogo consists of stacks of symbols: one stack for each position in the sequence. The overall height of the stack indicates the sequence conservation at that position, while the height of the symbols within the stack indicates the relative frequency of each amino acid at that position. Amino acids are coloured according to their chemical properties: polar amino acids (G,S,T,Y,C,Q,N) are in green; basic (K,R,H) in blue; acidic (D,E) in red, and hydrophobic (A,V,L,I,W,F,M) amino acids are in black. Bit: measure of conservation at a particular sequence position; the maximum conservation for a given amino acid in a sequence is 4.32 bits.

As stated in the Background section, previously described deduced ST proteins [33-36] are characterized by the presence of a signal peptide, a mature N-terminal end before repeats, and tandem repeats of 25/26 amino acids (Figure 3A). According to Katti et al. [2], ST proteins belong to category II of proteins having repeats in tandem; that is, those with the repeats ranging in length from 20 to 40 residues. Thus, several ST protein characteristics will be discussed in comparison with previously described proteins of that group.

All ST mature proteins are highly hydrophilic, except SaeST1, which is hydrophobic at its C-terminal. Because it was the only exception, we consider that there was probably a sequencing mistake at the end of the coding sequence.

The charged amino acids (DEKRHYC) varied from 30 to 49%, 87% of the sequences ranging from 35 to 45%, and the percentage of acidic amino acids usually being higher than basic ones.

ST proteins display several patterns with high occurrence as N-glycosilation, several different phosphorylation, N-myristoylation and amidation sites. Furthermore, CcaST2 and MesST3 showed a cell attachment consensus sequence, GmaST2 and VunST2 a Lys-rich consensus pattern, and TveST2 a peptidase pattern as analyzed by the ScanProsite tool.

There is a sequence, CteST1, which did not fulfil the general pattern, although it exhibited a similar structure and hence will be considered as ST-like and no properly as ST.

#### ST proteins enter the secretory pathway owing to the presence of a signal peptide

The subcellular location of a protein is an important goal in determining its function. Analysis of the 110 sequences having the N-terminal ends with the SignalP 4.0 server [38] revealed that all deduced ST proteins had a signal peptide (Figure 3A), which indicates that these proteins enter the secretory pathway. Fifty-five of the sequences analyzed had the predicted cleavage site between positions 22 and 23 as determined using the SignalP 4.0 logarithm [38], cleavage sites 23/24 (16%) and 21/22 (12%) being well represented. The signal peptide ranged between 19 and 28 amino acids in length. It should be noted that 72% of the sequences had a score above 0.800 (0.450 is the lowest score for a sequence to be considered a signal peptide) [38], the highest being 0.941 (MesST3) and the lowest 0.539 (MtrST5), probably due to an incorrect deduced intron splicing. Once the cleavage site had been determined, we observed that the mature protein started with an Arg in all 120 sequences except in 2: BpeST2 and MtrST5.

The presence of that clear signal peptide in each sequence analyzed indicates that they enter the secretory pathway and could be targeted extracellularly, although the final destination must be studied further. Thus, a bioinformatic analysis of protein targeting was carried out with 72 full-length ST proteins on three servers: WoLF PSORT [39], PSORT [40] and the TargetP 1.1 server [41].

As expected, TargetP 1.1 clearly revealed that the proteins enter the secretory pathway (81% with a score higher that 0.700) but not chloroplasts, vacuoles or other locations, whose scores never reached 0.285, usually being lower than 0.100. The analysis of sequences by PSORT was more complex because the scores for vacuole were slightly higher than for outside the cell in 65% of the sequences (0.900 and 0.800, respectively) and in 26% the score for the outside prediction was higher (0.800 versus 0.400 approximately). In two sequences (HciST1 and RpsST1) we obtained a high score for the outside localization and no score for the vacuole; in another two sequences the score was much higher for the vacuole and, finally, two more sequences (TofST1 and PpeST2) gave a predicted location in the ER membrane, with a score of 0.280 and no targeting either to the vacuole or outside the cell. In all cases, low scores of around 0.100 were found for several other locations such as the lumen of the ER, the ER membrane, peroxisome, the mitochondrial outer membrane, the Golgi apparatus, the plasma membrane, and the nucleus. Finally, according to the WoLF PSORT prediction, 46% of ST proteins were clearly targeted to the extracellular compartment; 13% were not targeted to the outside, and 23% were targeted mainly to compartments other than the cell wall, such as the vacuole, chloroplast, ER and even the nucleus. The location of the remaining ST proteins could not be established since several locations had the same scores. It is noteworthy that in some cases the triple analysis gave different results for the same protein. Also, a multiple location of a given ST cannot be excluded.

Members of the ST family in a given plant species have different locations, which indicates different putative roles in plant physiology. For instance, the targeting prediction for the six STs of barrel medic was to chloroplasts for MtrST1, ER for MtrST3 and 4, extracellular for MtrST5 and 6, and extracellular, vacuole and ER locations with the same score for MtrST2.

A unique location is not a common characteristic for proteins bearing tandem repeats. In proteins with 20 to 40 amino acid repeats, locations in nucleosomes (WD-40) [42], chloroplasts (EPR and TPR) [25,43] and mitochondria (PPR) [44], among others, have been suggested.

From our data it is clear that ST proteins enter the secretory pathway and that several proteins will end up in the cell wall, although individual proteins could have different final targets, such as the vacuole. It should be noted that since the predicted isoelectric point (pI) of ST proteins is clearly acidic (93% of total mature full-length sequences), 71% ranging from 4.37 to 5.37, if they were located in the cell wall (pH around 5) the ionic interactions would be very weak and unless they interacted by covalent bonds no strong binding to the cell wall would be expected.

#### The mature N-terminal end has conserved characteristics

The N-terminal end before the repeats of ST proteins is defined as the area that extends from the end of the signal peptide to the start of the tandem repeat region. Its length varied considerably, ranging from 30 to 80 amino acids, except for HciST1 (19) and AfpST2 (114), the most frequent being from 50 to 60 amino acids (32%) (Figure 3B). This region starts with an Arg, which is the first amino acid of the mature protein, as previously indicated, followed in most cases by Lys (63%). Six amino acids (7 in PsaST2 and PgiST2) away from the first Arg, Tyr-Trp were found in highly conserved positions seven and eight in 83% of the sequences analyzed, this Trp being the only one in the mature protein, except in 5 sequences (VviST4, VviST5, VviST6, StuST2 and MesST3). In this region the only Cys of the mature sequence appeared. 16% of ST sequences lacked such Cys, and no other Cys was found in any part of the mature protein. Exceptions were found in CmoST2, MtrST4 and QroST1, where a Cys appeared instead of a conserved Tyr in the tandem repeats. This exception could be explained in terms of a putative mistake in sequencing because only one base differed between the Cys codon and the Tyr codon. Only MtrST6 lacked Tyr, Trp and Cys. No conserved distances were found between Tyr-Trp and Cys (the most frequent being 30 to 40 (53%) and 20 to 30 (37%)) or between Cys and the start of the first repeat oligopeptide (the most frequent being 10 to 20 (55%)). It seems that these distances, strongly associated with phylogenetic closeness, are not very important in the role of ST proteins.

#### Mature ST proteins have tandem repeat oligopeptides that share a hexapeptide followed by 4 partially conserved amino acids and a fully conserved tyrosine at position 11

The second region (C-terminal) in the mature protein is the tandem repeat oligopeptide area (Figure 3A). This region may start with 1 or 2 imperfect repeats that sometimes have different lengths or lack the characteristic of the canonical repeats. Finally, this region usually ends with a truncated repeat of 13 amino acids.

The repeats start with a common hexapeptide (E/D)FEPRP [(Glu/Asp)-Phe-Glu-Pro-Arg-Pro], variable in the first amino acid, which may be Glu or Asp. Four amino acids, designated X_4,_ and a fully conserved Tyr follow the hexapeptide [33,34,36]. Thus, after translation the sequences with at least one sequence similar to (D/E)FEPRPX_4_Y were considered ST proteins. Furthermore, each time this sequence was found, we decided that a new repeat had started, including the last truncated one.

Analysis of the putative 72 ST full-length sequences revealed considerable variability in the number of repeats, ranging from 2 (as in VviST4, 5 and 6) to 17 (as in MtrST4 and 6). In fact, those having only two repeats had a canonical repeat and a truncated one, giving the smallest ST proteins, with fewer than 142 amino acids. Thus, the number of repeats would be responsible for the different sizes of ST proteins. This variability has also been found in other 20 to 40 amino acid tandem repeat proteins, such as TPR proteins [45] and EPR proteins [25], indicating that along evolution the loss and gain of these units has occurred frequently [1,46]. The origin of tandem peptide repeats can be attributed to intragenic duplication and recombination [28], which could explain the differences in number but also the similarity in the repeats within a protein and within a genus and family, as discussed below.

The length of the repeats also varied, ranging from 12 amino acids (VunST1) to 34 amino acids (PgiST2). The repeats having 25 (36%), 26 (26%) or alternating repeats of 25 and 26 amino acids (7%) were clearly predominant (69% of the ST sequences). In fact, 89% of the sequences analyzed had repeats ranging from 22 to 27 amino acids. Most of the ST proteins (85%) had each regular repeat of the same size (except the starting one and the truncated one); 13% had alternating repeats of different size, and in three of them (MesST3, 4 and 5) each repeat was of a different size.

To detect common characteristics within the repeats, a comparison was made using only two repeats of each protein (Figure 3C) represented with the WebLogo application [47]. It was clear that the consensus sequence of 122 analyzed proteins (244 repeats) (see Additional file 3), provided the same result observed previously; that is DFEPRPX_4_Y. However, sometimes certain differences were observed in a given species, most frequently the substitution of Asp by Glu in the hexamer. Each repeat had one Tyr at position 11, except for a few sequences that had two Tyr in each repeat (CarST1, LcuST1, LjaST2, MtrST1, PgiST2, PsaST1, PraST1, RpsST1, SmiST2) separated by 4 to 12 amino acids, while others had 1 or 2, depending on the repeat (CcaST2, JcuST2, LjaST1, TveST2). In several tandem repeat proteins, such as TPR and PPR, a conserved Tyr is involved in maintaining the tertiary structure of these proteins (2 alpha amphipathic helices) by hydrophobic interactions [48]. In structural cell wall proteins, Tyr is able to form isodityrosine links, both inter- and intra-molecular, making a structural protein net [7]. Conserved Tyr could play a similar role in ST proteins.

The X_4_ in the consensus sequence could determine a putative N-glycosylation signal (analyzed by ScanProsite tool, http://prosite.expasy.org/scanprosite/), as we will discuss later on.

The establishment of a consensus pattern in the repeat from Tyr at position 11 is difficult due to the different lengths of the repeats. Therefore, only 25 and 26 amino acid repeats were used in that analysis. Thus, the general consensus sequence for each repeat was DFEPRPX_4_YX_6-7_KXKKXFXK (Figure 3D), which shows a less conserved Lys at position 19, 21, 22 and 26 (26 amino acids repeats) or 18, 20, 21 and 25 (25 amino acids repeats). Apart from the X_4_ positions, amino acids X_6-7_ in the consensus pattern are phylogenetically conserved, being rich in Asp and Asn.

The existence of highly conserved patterns in the repeats is infrequent among tandem repeat oligopeptide proteins, most of them having degenerated repeats with occasional conserved amino acids at fixed positions, probably involved in the three-dimensional structure [49]. There is one exception: EPR proteins [25] with a repeat of 25 amino acids and a X_2_CX_4_CX_10_CX_2_HGGG consensus sequence, which is clearly different from the ST proteins consensus sequence. The presence of perfect, or nearly perfect, tandem repeats could indicate the structural and functional importance of each residue or recent evolutionary events [46]. The degree of perfection of tandem repeats can be determined by means of a parameter, called *P*sim, that can be calculated with the T-REKS program [50]. *P*sim values equal to or higher than 0.7 indicates that the proteins have perfect (value 1) or nearly perfect tandem repeats [46]. Analysis of the 72 ST full-length proteins by T-REKS indicated that 72% had a *P*sim value higher than 0.7, 33% being higher than 0.8 and 1% (sequence OcsST2) higher than 0.9. Jorda et al. [46] found that the level of repeat perfection was correlated with their tendency to be unstructured.

Although to date no studies on ST tertiary structure have been performed, the presence of many conserved tandem repeats seems to point to a slight tendency to form a single, well-defined structure. By contrast, others defined proteins with tandem repeats of 20 to 40 amino acids are proteins with a well-established three-dimensional structure, often involved in mediating protein-protein interactions, as indicated in the Background section.

#### ST proteins are characterized by a DUF2775 domain

Most of the ST deduced proteins studied, 134 out of 136, have one or several complete and/or partial Domains of Unknown Function, DUF2775 [51,52], Pfam family 10950 [53], that do not extend to the signal peptide present in those proteins, as will be discussed below. DUF2775 was defined in the basis of 8 ST sequences acting as a core (http:pfam.sanger.ac.uk/family/DUF2775). The multiple sequence alignment shows a highly conserved motif of 135 amino acids. This motif comprises approximately 20 amino acids from the non-repeating area of the peptide, 2 tandem repeats and 1 truncated tandem repeat. DUF2775 appeared in all proteins, even though they were not full-length ones. RhiST2 and FanST2 sequences, from two genera of the Rosaceae family were the only exceptions, with a slightly different structure, although it is not clear whether there might be a phase shift in the translated sequence. This DUF2775 domain is a specific characteristic of ST proteins and it has not been described in other type-II tandem repeat proteins [2]. All ST proteins have at least one DUF2775 domain and they may also have other truncated DUF2775 domains ranging in number. As DUF2775 contain 3 tandem repeats, they can be overlapped in the same protein.

According to BLAST, 47% of the full-length sequences had only one DUF2775 domain while 30% had one complete domain and one or several (up to five in MtrST4 and MtrST6) truncated DUF2775 domains. The highest number of complete DUF2775 domains in one sequence was 3, found in MtrST1, BpeST1 and CarST2, this latter also having a truncated domain. Several sequences had overlapping DUF2775 domains, such as SaeST2, TprST2, LjaST1, AmaST1 and AglSt2, and others had only truncated DUF2775 domains (14%), as in HarST2, HcuST1, HciST1 and TofST1.

#### Three main types of ST proteins can be defined according to their putative glycosylation pattern

As stated above, the X_4_ in the consensus sequence repeats was conserved among phylogenetically close species. The analysis of X_4_ in all 135 sequences (except CteST1 with X_5_) showed that 84% (114 sequences) predominantly had one kind of consensus pattern, while 16% had a mixture of different patterns, the mixture of NxSx and DxT/Sx (29%) being predominant.

**Figure 4:**
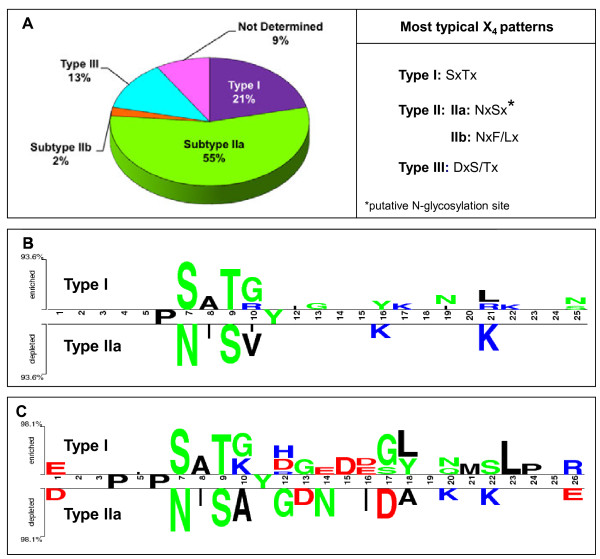
**Identification of three main types of ST proteins according the X**_**4**_**pattern.** (**A**) Percentages of the different types of ST proteins and the most typical X_4_ pattern found in each type. (**B, C**) Comparisons between type I and type IIa ST proteins represented using Two Sample Logo. (**B**) Comparison of 25 amino acids repeats of type I versus type IIa. The main difference is found in the X_4_ sequence. (**C**) Comparison of 26 amino acids repeats of type I versus type IIa. The difference in the conserved X_4_ sequence and the preference for D in type IIa in the first amino acid of the hexamer could be noted. The repeats of 26 amino acids showed greater variations, probably due to the higher sequence number analysed. The Two Sample Logo consists of stacks of symbols: one stack for each position in the sequence, the upper part represents type I proteins preference for one amino acid in a given position with respect to type IIa amino acid at the same position and vice versa in the lower part. The overall height of the stack indicates the sequence conservation at that position, while the height of symbols within the stack indicates the relative frequency of each amino acid at that position. Amino acids are coloured according to their chemical properties: polar amino acids (G,S,T,Y,C,Q,N) are in green; basic (K,R,H) in blue; acidic (D,E) in red, and hydrophobic (A,V,L,I,W,F,M) amino acids are in black.

The X_4_ sequence determines the presence or absence of one N-glycosylation site per repeat and could indicate different types in the ST protein family. Thus, the X_4_ pattern could be used to identify three main ST types (Figure 4A):

Type I. This type represents 21% of ST proteins and are characterized by sequences with Ser at position 7 in most repeats (20%, the consensus sequence being SxTx (x usually being Lys, Val, Ala or Gly). These repeats do not have any N-glycosylation site between the hexapeptide and the Tyr at position 11. In each species, only one type I ST was found, and this sequence was designated ST1 in each species. Sequences with an X_4_ pattern other than SxTx were designated ST2 (and following), even when only one sequence was found in the species in question.

Type II. Characterized by sequences with Asn at position 7 in most repeats. This type includes two subtypes: IIa, where the X_4_ sequence is NxSx (x usually being Ile, Val, Ala) with an N-glycosylation site in all or almost all repeats, and IIb, where X_4_ is Nx(F/L)x (x usually being Phe, Ser, Ala or Leu), without an N-glycosylation pattern. Subtype IIa was the most abundant (55% of ST proteins) and several members of this subtype could be found within one species, such as PsaST2, 3 and 4 in *P. sativum*, or MtrST2, 3, 4 and 5 in *M. truncatula*. Only three ST proteins, representing 2% of the STs, belonged to subtype IIb: MtrST6, TprST2 and PpeST2. Whether the type IIa sequences might be subject to a true N-glycosylation and the role of such post-translation modifications in the function of the protein remain to be determined empirically.

Type III. Characterized by sequences having Asp or Thr at position 7 in most repeats. This type represents 13% of ST sequences, X_4_ being often DxT/Sx. As in type I and IIb, these repeats do not have any N-glycosylation site between the hexapeptide and Tyr at position 11.

There are also ST proteins with a mixture of X_4_ patterns in the different repeats that usually cluster in one of these types, depending on the predominant pattern.

The differences between the more abundant protein types, I and IIa, can be visualized by Two Sample Logo [54] (Figure 4B,C). It was observed that the main difference was the X_4_ sequence in both, 25 (Figure 4B) and 26 (Figure 4C) amino acids repeat comparison. Also, in some type I ST a second Tyr can be found. The repeats of 26 amino acids showed higher variations (Figure 4C), probably due to the higher sequence number analyzed. A preference for Asp in type IIa at several positions, including the first amino acid in the hexamer could be noted (Figure 4C). In contrast some positions are fully conserved in type I and IIa STs, Pro in position 6 and Tyr in position 11 (Figure 4B,C). Furthermore 26 amino acid comparison showed an extra fully conserved Pro at position 4 (Figure 4C).

**Figure 5:**
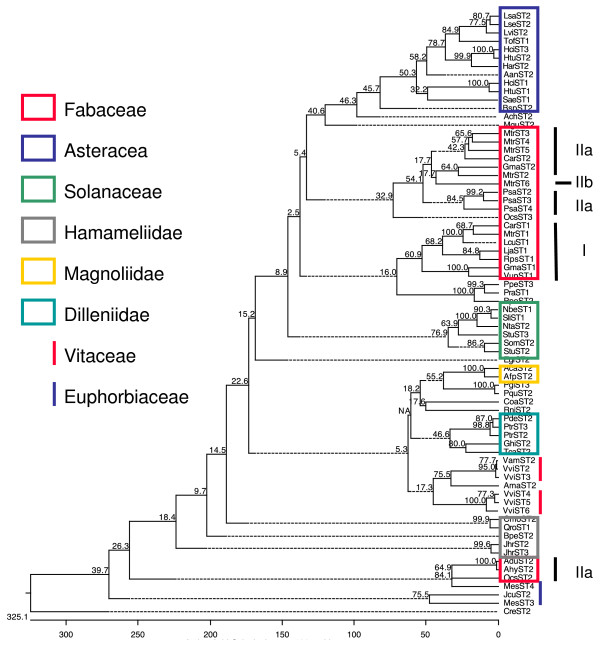
**Phylogenetic tree of ST sequences.** A phylogenetic tree using the MegAlign program from DNASTAR® Lasergene 10.0 software, applying the CLUSTAL W program and a bootstrap (n= 2000) analysis, was carried out using 72 mature full-length ST sequences. Sequences clade according to taxonomic subclass and even to families. ST proteins from the Fabaceae family split into two groups, and a clear separation in the established types of ST proteins can be seen.

To check whether these different types could indeed group sequences with common characteristics, a phylogenetic tree was generated using the 72 mature full-length sequences (Figure 5). It was observed that the sequences claded according to taxonomic subclass, and even families, which indicates that several characteristics (such as the N-terminal region, the number and length of repeats, etc.) are related to the taxonomic group. ST proteins from the Fabaceae family split into two groups, and a clear separation among the above types was observed. However, it is clear that not only the X_4_ pattern contributed to the grouping of the proteins, since the taxonomic characteristics in the less conserved part of the repeat were also important to determine phylogenetic tree clades.

### ST proteins are neither known cell wall structural proteins nor arabinogalactan proteins

As mentioned above, all ST proteins had a signal peptide that targets them to the secretory pathway and, perhaps, to cell walls. Additionally, several cell wall proteins have repeats of short oligopeptides in which amino acids with important functions, such as Pro, Tyr and Glu, are present. Taking this into account, a comparison between ST proteins and structural and arabinogalactan cell wall proteins was made in order to determine whether ST proteins might be a previously characterized cell wall protein type.

In this comparative analysis, the GRP protein group was ruled out directly, since the content of Gly in GRP was higher than 28%, usually around 40-75%, while in ST proteins the Gly percentage was below 11%. Additionally, Pro was not found within the repeat sequences in GRPs, unlike STs.

Structural proteins such as HRGPs and PRPs are more similar to STs and present conserved regions with repeat sequences rich in amino acids such as Pro, Ser, Val, Tyr and Lys [4,5].

**Figure 6:**
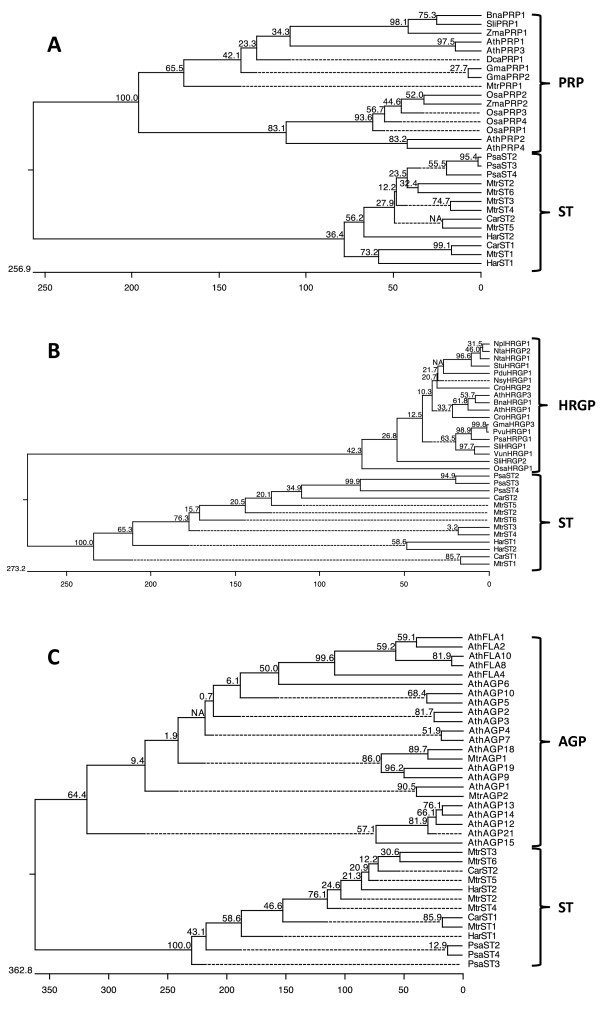
**Comparison between cell wall structural proteins and arabinogalactan proteins and ST proteins.** Phylogenetic trees were constructed as indicated in Figure 5. **A**. Phylogenetic tree constructed with different PRP types and ST protein sequences. **B**. Phylogenetic tree constructed with different HRGP and ST protein sequences. **C**. Phylogenetic tree constructed with different AGPs types and ST sequences. In all cases, STs appear as a clearly separate cluster. PRPs accession numbers (alphabetically): *Arabidopsis thaliana* AthPRP1 [UniProtKB:Q9M7P1], AthPRP2 [UniProtKB:Q9M7P0] AthPRP3 [UniProtKB:Q9M7N9] and AthPRP4 [UniProtKB:Q9M7N8]; *Brassica napus* BnaPRP1 [UniProtKB:Q39353]; *Daucus* carota DcaPRP1 [UniProtKB:Q39686]; *Glycine max* GmaPRP1 [UniProtKB:P08012] and GmaPRP2 [UniProtKB:P13993]; *Medicago truncatula* MtrPRP1 [UniProtKB:Q9FEW3]; *Oryza sativa* OsaPRP1 [UniProtKB:Q94H12], OsaPRP2 [UniProtKB:Q7GBX3], OsaPRP3 [UniProtKB:Q94H10] and OsaPRP4 [UniProtKB:Q7XGS2]; *Solanum licopersicum* SliPRP1 [UniProtKB:Q00451]; *Zea mays* ZmaPRP1 [UniProtKB:Q41848] and ZmaPRP2 [UniProtKB:Q9SBX4]. HRGPs accession numbers (alphabetically): *A. thaliana* AthHRGP1 [UniProtKB:Q38913] and AthHRGP3 [UniProtKB:Q9FS16]; *B. napus* BnaHRGP1 [UniProtKB:Q8LK15]; *Catharanthus roseus* CroHRGP1 [UniProtKB:Q39599] and CroHRGP2 [UniProtKB:Q39600]; *G. max* GmaHRGP3 [UniProtKB:Q39835]; *Nicotiana Plumbaginifolia* NplHRGP1 [UniProtKB:Q40402]; *Nicotiana sylvestris* NsyHRGP1 [UniProtKB:Q9FSG0]; *Nicotiana tabacum* NtaHRGP1 [UniProtKB:Q40503] and NtaHRGP1 [UniProtKB:Q06802]; *O. sativa* OsaHRGP1 [UniProtKB:Q40692]; *Phaseolus vulgaris* PvuHRGP1 [UniProtKB:Q09083]; *Pisum sativum* PsaHRGP1 [UniProtKB:Q9M6R7]; *Prunus dulcis* PduHRGP1 [UniProtKB:Q40768]; *S. licopersicum* SliHRGP1 [UniProtKB:Q09082] and SliHRGP2 [UniProtKB:Q09084]; *Solanum tuberosum* StuHRGP1 [UniProtKB:Q06446]; *Vigna unguiculata* VunHRGP1 [UniProtKB:Q41707]. AGPs accession numbers (alphabetically): *A. thaliana* AthAGP1 [UniProtKB:Q8LCN5], AthAGP2 [UniProtKB:Q9SJY7], AthAGP3 [UniProtKB:Q9ZT17], AthAGP4 [UniProtKB:Q9ZT16], AthAGP5 [UniProtKB:Q8LCE4], AthAGP6 [UniProtKB:Q9LY91], AthAGP7 [UniProtKB:8LG54], AthAGP9 [UniProtKB:Q9C5S0], AthAGP10 [UniProtKB:Q9M0S4], AthAGP12 [UniProtKB:Q9LJD9], AthAGP13 [UniProtKB:Q9STQ3], AthAGP14 [UniProtKB:Q9LVC0], AthAGP15 [UniProtKB:Q9LYF6], AthAGP18 [UniProtKB:Q9FPR2], AthAGP19 [UniProtKB:Q9S740], AthAGP21 [UniProtKB:Q9C8A4], AthFLA1 [UniProtKB:Q9FM65], AthFLA2 [UniProtKB:Q9SV13], AthFLA4 [UniProtKB:Q9SNC3], AthFLA8 [UniProtKB:O22126] and AthFLA10 [UniProtKB:Q9LZX4]; *M. truncatula* MtrAGP1 [UniProtKB:G7K3Y3] and MtrAGP2 [UniProtKB:G7JV60].

Comparison of the different PRP types and ST proteins led to the conclusion that ST is not a new type of PRP protein, as clearly shown in the phylogenetic tree represented in Figure 6A, in which STs appear as a separated cluster. There is one common characteristic among them: the content of Tyr (ranging between 1.5 and 8.3% in PRP types and from 3.2 to 7.2% in STs) and its conserved position. In PRPs, Tyr residues allow the formation of linkages by peroxidase-mediated oxidative coupling between Tyrs, Tyr and lignin, and/or Tyr and pectic polymers. These linkages are able to interconnect structural cell wall elements [7,55]. The presence of conserved Tyr in ST proteins suggests a possible structural role in the cell wall. However, theoretically ST proteins cannot form intramolecular isodityrosine or diisodityrosine linkages, since their formation requires consecutive Tyr [7,55]. We cannot discard the possible formation of intermolecular bridges, although ST proteins do not present the characteristic motifs described for this: VYK, YSY, YYY or YKYK. Furthermore, several differences were observed between PRP and ST proteins. One fundamental difference was seen in the way that the repeats are arranged in the total protein sequence in PRPs and STs. PRPs had different repeats of 3 to 7 amino acids along the protein sequence, depending on the PRP type: PPxx (K/T) (x = V, Y, H, E); PPYV; PPTPRPS; PPV, P(V/I)YK, KKPCPP; PEPK; PKPE, P(V/E)PPK. These small repeat peptides could be comparable to the characteristic hexapeptide of STs. However, the PRP repeats are not included in a larger conserved domain, as happens in STs, but dispersed or arranged in tandem or in clusters in the protein sequence. Another discrepancy is the pI, which was much higher in PRPs (between 8 and 10) as a consequence of the lower percentages (below 12%) of acidic amino acids, while in the STs this percentage sometimes reached 26%, and the pI, with the above-mentioned exceptions, was acidic (predominantly below 5). A notable difference was also found in the Pro content, between 16 and 36% in PRPs, while in STs it was lower than 11%.

The phylogenetic tree constructed with HRGP and ST sequences indicated that both proteins were also very different (Figure 6B) and, as in the case of PRP, we found several different characteristics in protein composition, pI (9.8 for HRGPs), the content in acidic amino acids (0.7% in HRGP and a 20% on average for STs) and the percentage of Pro (44% in HRGP; below 11% in ST) and Tyr (15% in HRGP; below 7% in STs), which are two important amino acids in the structure of these proteins.

The above differences found among STs and both PRP and HRGP indicate that STs cannot be included in these structural protein groups and the bootstrap analysis also indicated that STs are evolutionarily even more distant from HRGPs than from PRPs.

The phylogenetic tree constructed with AGPs and STs confirms the separation of both types of protein. Evolutionarily, AGPs are more separated from ST than PRPs and HRGPs (Figure 6C). Several differences were also observed in amino acid composition, excluding fasciclin-like AGPs (AthFLA in the figure). The content in acidic amino acids (5% on average in AGPs and 20% in STs) and the percentage of Tyr (0% in AGPs; higher than 3% in STs) were the clearest differences. Moreover, classic AGPs and Non-GPI AGPs had high Pro percentages (27% and 21%, respectively) in comparison with ST (8%), and AG peptides (AthAGP12, 13, 14, 15 and 21) had a fairly low molecular weight (4 kDa). The fasciclin-like type was the AGP class most similar to STs upon taking into account the molecular weight, the pI and the amino acid composition, with similar percentages of Pro (7%); the content of Tyr (2.3%) and acidic amino acids (9.2%) was lower than in ST proteins but higher than for other AGPs.

Furthermore, the conserved regions of AGP are mainly formed by glycomodules, repeats of Ala-Pro, Ser-Pro and/or Thr-Pro, where most of the Pro (up to 85%) is hydroxylated and Ala-Pro and Ser-Pro are almost always hydroxylated and O-glycosylated [56]. No such glycomodules were found in ST proteins, pointing to a significant difference with AGP.

In conclusion, if located extracellularly ST proteins are a class of cell wall proteins different from any known structural cell wall proteins and from arabinogalactan proteins. Furthermore, ST proteins belong to tandem repeat proteins of 20 to 40 amino acids, unlike all other cell wall protein types that belong to proteins with repeats from 2 to 20 amino acids.

### *ST* mRNAs accumulate mainly in roots and under biotic interactions

Study of the sequences appearing in EST databases provides information about the organs and the growth conditions of the plants from which the cDNA library was constructed. Analysis of such information allows us to know where an *ST* mRNA is accumulating or is predominant, although the fact that the gene could also be expressed in other organs or under other growth conditions cannot be discarded.

#### ST mRNAs accumulation in plant organs

Forty-nine percent of the ST sequences (149 out of 306 whose mRNA origin is known) were found in cDNA libraries made from mRNA isolated from the roots or radicles of 31 different species, most of them in *C. arietinum* (36 out of 39) and *M. truncatula* (47 out of 54) (Table 2). This percentage could be even higher, since 20% of the sequences (61) were isolated from mRNA obtained from a mixture of organs or whole plants. The sequences obtained in seeds (7.8%), leaves (7.2%) and elongating organs such as epicotyls, hypocotyls and stems (5.6%) were also significant. *ST* mRNAs have also been found in other organs, such as flowers, fruits, haustoria, buds, etc., with percentages lower than 2.6% (Table 2).

**Table 2:** **Distribution of *ST *mRNA sequences in different plant organs or tissues**

**Tissue/Organ**	**Total sequences**
Root and radicle	149
Stem, epicotyl and hypocotyl	17
Leaf	22
Flower and Flower bud	5
Seed, embryo, cotyledon	24
Fruit	8
Meristematic tissue	4
Tuber and rhizome	2
Haustorium	2
Callus	2
Trychome	3
Cambium and Bark	4
Primary xylem and phloem	1
Fiber	2
Mixture^’^	61
No indicated	21

The table indicates the plant organ or tissue used to extract the mRNA used to construct the cDNA library where the *STs* were found. Data were obtained from Additional file 1 (327 sequences). Mixture: different combinations of organs, tissues and/or whole plants/seedlings.

Analysis of the largest *ST* family, the barrel medic family, revealed that 5 of the six *STs* (except *MtrST5*) were present in roots. *MtrST3* was also found in immature seeds and *MtrST2* and *4* also in leaves. *MtrST5* was not found in any cDNA library.

Apart from the origin of the mRNA used to make the libraries, little is known about ST location. Most *ST* EST findings come from massive EST analyses, but without further analysis [57-59]. As an exception, in watermelon *ClaST2* transcript accumulation (studied by analytical techniques) is several times higher in green fruit as compared to leaves, decreasing as maturation progresses [60,61]. Waters et al. [62] and Fernández et al. [63] reported a similar transcript accumulation pattern for grape berry *VviST2*, with a maximum in the green stage, proposing a role of ST proteins in morphogenesis. *VviST2* transcripts were also found in roots and, to a lesser extent, in stems and leaves.

The accumulation of *ST* transcripts in different organs determined by Northern blot analyses has been studied in depth in *P. sativum*[33,35] and *C. arietinum*[34,37]. *ST* transcripts were detected in epicotyls and stems, either specifically (*PsaST2*) or not (*CarST*). In chickpea, transcript accumulation was higher in radicles than in epicotyls, whereas in garden pea mRNAs were not detected in roots. In fact, in the chickpea transcriptome database (CTDB, http://www.nipgr.res.in/ctdb.html) developed by Garg et al. [64] the expression profile of *CarST1* (TC06623) and *CarST2* (TC01499) indicated a higher transcript accumulation of both genes in roots than in shoots (about 12 fold). In chickpea, no expression was found in mature leaves, flowers, young pods or immature seeds [34,64]. A pod-specific ST (*PsaST3*) was described in garden pea [36], in which no other *ST* transcripts were found in cotyledons, leaves and roots. However, *PsaST2* and *PsaST3* mRNAs were isolated in cDNA-specific libraries from leaves (Additional file 1), indicating that their mRNAs were present, although probably at such low levels that they could not be detected by Northern blot analyses [33,36].

#### ST mRNAs accumulation in plants under biotic and abiotic stress conditions

Regarding plant growth conditions, while 184 sequences (56.3%) came from plants growing under optimal conditions of light, temperature and humidity and in 21 the mRNA origin was unknown, 122 sequences (37.3%) came from plants growing under stress conditions or under chemical treatment, most of them under biotic interactions (21%) with pathogenic and mycorrhizal fungi (7.3 and 4.6%, respectively), pathogenic and N_2_-fixing bacteria (0.3 and 3.4%, respectively), nematodes (4.0%), parasitic plants (0.6%), insect (0.6%) or viruses (0.3%) (Table 3). They have also been found in plants under abiotic stresses (15%) such as salinity/drought stress (10.0%), Pi or N_2_ starvation (0.3 and 3.4%, respectively), N_2_ fertilization (0.6%), high Pi (0.6%), cold acclimation (0.3%) and hypoxia (0.3%) (Table 3).

**Table 3:** **Distribution of*ST *mRNA sequences under different growth conditions**

**Growth conditions**	**Total**
Optimal growth conditions	184
Pi starvation^(a)^	7
High Pi^(a)^	1
N_2_ starvation^(a)^	3
N_2_ fertilization^(a)^	2
Salinity/Drought stress^(a)^	34
Cold acclimated^(a)^	1
Hypoxia^(a)^	1
Insects interaction^(b)^	2
Pathogen Fungus interaction^(b)^	15
Mycorrhizal Fungus interaction^(b)^	24
Pathogen Bacteria interaction^(b)^	1
N_2_ –fixing Bacteria interaction^(b)^	11
Virus interaction^(b)^	1
Nematodes interaction^(b)^	13
Parasitic plants interaction^(b)^	2
Strigol treatment^(c)^	1
Giberellic acid treatment^(c)^	1
Salycilic acid treatment^(c)^	1
Benzothiadiazole treatment^(c)^	1
Not indicated	21

The table indicates the growth conditions of the plant used to extract the mRNA from which the cDNA library, containing *ST* sequences, was constructed. (a) abiotic stress; (b) biotic interactions; (c) chemical treatment. Data were obtained from Additional file 1 (327 sequences).

Although the isolation of *ST* sequences in cDNA libraries does not necessarily mean that *ST* genes are expressed as a consequence of biotic or abiotic interactions, the high percentage (39%) (Table 3) of *ST* sequences found in plants under such stresses indicates that *ST* mRNAs are abundant in these growth conditions.

It is possible that stress could up- or down-regulate the expression of *ST* genes, without complete induction or repression. For instance, as can be seen in Additional file 1*VviST3* was isolated from both GA_3_-treated and -untreated fruits, *Casuarina glauca ST2* (*CglST2*) from inoculated and uninoculated *Frankia* roots, *Phaseolus vulgaris ST1* (*PvuST1*) in roots growing at high and low Pi, *G. max* (*GmaST1*) with and without several stresses, and *GmaST2* after *Heterodera* infection, but also in uninfected roots.

Moreover, the possibility that different *ST* genes might be expressed in different growth conditions, even under different biotic interactions, cannot not be ruled out. Focusing again on the barrel medic family (Table 4), *MtrST2* and *3* were the only two found under optimal growth conditions; *MtrST4* was the only one in leaves under insect (*Spodoptera exigua*) attack, and *MtrST2* was only found in a nematode (*Meloidogyne incognita*) interaction. Furthermore, *MtrST6* was only found in cDNA libraries constructed from mRNA extracted from roots under arbuscular mycorrhizal interaction and its transcripts do not appear under other microbe interactions [65]. In fact, *MtrST6* does not undergo changes in its expression pattern during systemic defence response although it does when establishing symbiosis [66,67], its transcript being strongly reduced when the formation of arbuscular mycorrhiza-induced apocarotenoids was inhibited [68], and increased when Myc factors were added to the medium [69]. *MtrST6* has been considered an early marker of mycorrhization [70], being up-regulated in both arbuscule-containing and non arbuscule-containing cells of mycorrhizal roots, although at a higher level in the former [71]. *MtrST1* transcripts are more abundant in uninfected roots than in roots with mature nodules [66], although they have never been found in cDNA libraries from optimal growth conditions. It is important to note that *A. thaliana*, one of the few species that are non-mycorrhizal, does not have *ST* genes.

**Table 4:** **Distribution of the *MtrST *mRNA sequences according to growth conditions**

	***MtrST1***	***MtrST2***	***MtrST3***	***MtrST4***	***MtrST5***	***MtrST6***
Optimal growth conditions	----	2	5	----	----	----
Mycorrizal interaction	4	1	----	----	----	16
N_2_-fixing interaction	2	----	1	1	----	----
Insect attack	----	----	----	2	----	----
Nematode interaction	----	5	----	----	----	----
Pathogenic interaction	----	6	----	1	----	----
Pi Starvation	1	2	----	2	----	----
N_2_ starvation	----	1	1	1	----	----

Six different *Medicago truncatula ST* sequences (*MtrST*) were found. Numbers indicate the number of times a specific sequence was found in each growth condition. The specific interaction can be found in Additional file 1.

Thus, it is possible that proteins encoded by *ST* genes, called ST proteins, have specific roles in a given interaction but also that some of them could act in several biotic stresses in different tissues, such as MtrST2 and MtrST4 for example (Table 4). Bearing in mind that some genes expressed in pathogen infection can also be expressed in the initial process of symbiosis [72], and since both processes activate defence mechanisms in plants involving molecular interactions between two organisms, it can be proposed that several ST proteins might act in both processes. Also, it has been reported that some genes are essential for the development of symbiosis with mycorrhizal fungi and with N_2_-fixation bacteria [73,74]. In this sense, we found *ST* mRNA sequences in N_2_-fixing initial phases in symbiosis with *Rhizobia*, such as in *Lotus japonicus* with *Mesorhizobium loti*, *M. truncatula* with *Shinorrizobium meliloti* and *G. max* with *Bradyrhizobium japonicua* and with mycorrhizal fungi, as in the interaction of *M. truncatula* and *Glomus versiforme.*

A special kind of biotic relationship is parasitism. In this sense, *ST* mRNAs have been isolated from the parasitic plants *Striga asiatica* and *Tryphysaria versicolor* when they were forming haustoria [75,76]. This points to a possible function in the interaction between a parasitic plant and its host and strengthens the notion of a general role in interactions between organisms.

Some function in abiotic stress could also be played by ST proteins. In fact, as well as in optimal growth conditions [34] chickpea *ST* mRNAs have been found in the roots of drought- and salinity-stressed plants [77]. However, *CarST1* and *CarST2* transcript accumulation decreases under abiotic stress, the strongest effect being under salinity and low-temperature treatment [34].

Few reports can be found concerning the involvement of ST proteins in physiological processes other than biotic or abiotic stress responses and the previously discussed role in morphogenesis and growth. As an example, in *P. persica PpeST2* has been reported to be the most up-regulated gene in the late adaptation to bud dormancy induced by short days, while no transcript increase was observed in a mutant without dormancy [78].

Many more studies must be done to determine the actual role of ST proteins in plant physiology. The analysis of mRNA accumulation points to two possibilities: one, that these proteins play a common role in several processes, and two, that different members have specific roles in different processes.

## Conclusions

We have described a new family of proteins called ST with, at least to date, 136 members. Their presence is limited to the plant kingdom, specifically to a few families of dicotyledonous plants. A high percentage of the sequences similar to ST found in databases come from libraries of cDNA from radicles and roots of plants growing under biotic and abiotic stress. They have 20 to 40 amino acid tandem repeat sequences with differential characteristics from the reported group of 20 to 40 amino acid tandem repeat proteins (signal peptide, the DUF domain, conservative repeats regions) and also from known cell wall proteins with repeat sequences such as PRP, HRGP, GRP and AGP.

The data obtained by our EST analyses, as well as empirically [65,67,69-71], relate ST proteins to biotic interactions as their possible involvement in symbiosis and abiotic stress. A member of the ST family has been related to early fruit morphogenesis both in grape berry [62,63] and in watermelon [60,61], although its exact role in such processes remains elusive. The first studies by De Vries et al. [33] and Muñoz et al. [34] suggested a role in plant cell elongation in organ growth not related to light, the action of auxin and/or brassinolides, or to germination [37]. The similarity to seed storage proteins has been discarded for PsaST2, although these proteins present a certain homology [33]. Different STs could play different roles in plant development.

ST proteins are not a shoot/stem-specific proteins nor tissue-specific, as their name initially indicated, being more related to roots, although different family members could have different locations within the whole plant and could play different roles in development. In fact the original name (**S**hoo**T** specific protein) is confusing for the identification of the family members.

## Methods

### Search for ST-like nucleotide sequences

In order to find ST nucleotide sequences, we performed different types of searches for sequence similarity using the FASTA program [79] at the EMBL-EBI web pages ( http://www.ebi.ac.uk/Tools/), initially employing chickpea ST sequences [EMBL: X97454 and EMBL:X97455).

Thus, full-length ORFs of chickpea *CarST1* and *CarST2* nucleotide sequences were used as well as the nucleotide sequences of their conserved repeats (75 to 78 nucleotides). The first search was done against the EMBL Release, which includes databases of all living organisms. All sequences found were plant sequences, and hence a new search was performed using only EMBL Plant, which includes EMBL EST (Expressed Sequence Tag, cDNA fragment sequences), with a score of 1000 results. A third search was done using members of all taxonomic families. Furthermore, once we had at least one sequence from each species in which an ST sequence appeared, we repeated the similarity search with that ST sequence at the EBI web page to look for the largest fragment for each species in the databases.

Most of the ST sequences obtained in the above searches, checked for chickpea ST characteristics as explained below, are summarized in Additional file 1. A 3-letter code was used to describe the species because a 2-letter code was clearly insufficient since 29 species could not be identified clearly.

Finally, the *CarST1* and *CarST2* nucleotide sequences were used to check for *ST* genes in the genomes of the 25 species included at the v7.0 phytozome web page ( http://www.phytozome.net/), which provides comparative genomic studies among green plants. The sequences found were analyzed and selected as described above. The *ST* sequences found are summarized in Table 1.

### Translation into amino acid sequences and selection of ST proteins

All nucleotide sequences obtained were translated into the six possible phases either using the DNASTAR software from Lasergene (USA) or the ExPASy translate tool ( http://web.expasy.org/translate/), looking for characteristic ST repeats in the deduced proteins. These amino acid sequences were checked at the NCBI BLAST site ( http://www.ncbi.nlm.nih.gov/Blast.cgi) in order to determine the presence of the DUF2775 domain, present in all ST proteins. The sequences that did not have characteristic repeats or at least one complete or partial DUF2775 domain were discarded and the nucleotide sequences were not considered as ST, except in two cases.

#### Search for full-length proteins

In order to determine whether the EST fragment encoded a full-length protein or not, we checked the N- and C-terminal ends of the encoded amino acid sequence. Careful exploration of such borders also allowed us to determine whether there was the same or different ST protein. Thus, Additional file 3 contains a list of all the deduced sequences, indicating which ones were complete ST proteins, N-terminal complete, C-terminal complete, or merely an internal fragment.

The presence of a stop codon before the first methionine, a signal peptide at the N-terminus, as well as the amino acids Tyr-Trp at nucleotide 26–30 from first methionine, were taken into account to determine whether the deduced protein was a complete N-terminal sequence. It was more difficult to determine whether the C-terminal end of the protein was complete. First, the presence of a stop codon was analyzed, since there was the possibility of a phase shift in the sequence. However, the nucleotide and the amino acid sequences sometimes finished at the same point without a stop codon, and hence it was not possible to check further. In addition, most of the C-terminal ends of known full-length proteins ended with Tyr followed by two or three amino acids. Finally, the presence of undetected phase shifts cannot be ruled out. Taking all this into account, it was considered that a protein was complete at the C-terminal end if there was a stop codon in the translated sequence without a detectable phase shift and also when it ended with Tyr followed by two or three amino acids. In the remaining deduced proteins it was difficult to know whether the protein was complete at the C-terminal end or not.

### Phylogenetic tree of species having ST sequences

To visualize the phylogenetic distribution of all species having ST proteins a tree has been performed using the Common Tree Taxonomy tool at NCBI ( http://www.ncbi.nlm.nih.gov/Taxonomy/CommonTree/wwwcmt.cgi) according to the NCBI taxonomy database.

The tree was done with all species having ST proteins (listed in Additional file 2) as well as sixteen species without ST proteins whose genome has been fully sequenced as appeared in Phytozome v7.0. The species without ST sequences used were two green algae (*Volvox carteri*, *Chlamydomonas reinhardtii*), one moss (*Physcomitrella patens*), one lycophyte (*Selaginella moellendorffii*), five monocots (*Zea mays, Oryza sativa, Sorghum bicolor, Brachypodium dystachion, Setaria italica*) and six dicots (*Linum usitatissimum, Arabidopsis thaliana, A. lyrata, Brassica rapa, Thellungiella halophila, Carica papaya)*.

The tree generated was edited using FigTree v1.3.1 ( http://tree.bio.ed.ac.uk/software/figtree/).

### Bioinformatic characterization of ST proteins

#### Conserved domains and N-glycosylation pattern

All ST proteins were analyzed again using the CDD algorithm [51,52] in the BLAST database to determine the presence of DUF2775 conserved domains. However, only the proposed full-length sequences were used to determine the number of DUF2775 domains and the presence of other conserved domains.

The number of putative N-glycosylation sites was also determined using the PROSITE web page ( http://prosite.expasy.org/).

#### Subcellular localization

The sequences of the ST proteins were first analyzed on the CBS web page ( http://www.cbs.dtu.dk/index.shtml) using the SignalP 4.0 programs [38] ( http://www.cbs.dtu.dk/services/SignalP) to determine the putative presence of a signal peptide and TargetP 1.1 [41] ( http://www.cbs.dtu.dk/services/TargetP) to establish the subcellular location of the proteins. The first program was used with 110 protein sequences with N-end terminus while the second program was used in 72 full-length proteins. Once the presence of a signal peptide had been confirmed, its length was determined in order to carry out comparisons among mature proteins.

To confirm the results obtained as indicated above, all the protein sequences were also checked with the PSORT [40] and WoLF PSORT [39] ( http://wolfpsort.org/) databases, which allowed us to determine their predicted subcellular localization.

#### Biochemical characteristics and phylogenetic tree

Comparative analysis of the selected proteins was carried out with the DNASTAR Lasergene 10.0 software (USA).

The PROTEAN program was used to analyze the amino acid composition, hydrophobic profile, molecular weight and pI.

Alignments were done using the MegAlign program for multiple sequence alignment with the CLUSTAL W algorithm, using a bootstrap analysis of n=2000 for phylogenetic tree construction.

#### Characterization of tandem repeats

The T-REKS algorithm ( http://bioinfo.montp.cnrs.fr/?r=t-reks, [50]) was used to analyze the perfection of the tandem repeat sequences.

In order to analyze ST repeats, WebLogo ( http://weblogo.berkeley.edu/) and Two Sample Logo (TSL) ( http://www.twosamplelogo.org/) were employed as graphical representation for multiple sequence alignment of conserved positions of the amino acid in the repeats [47]. Different alignments were conducted with CLUSTAL W ( http://www.ebi.ac.uk/Tools/msa/clustalw2/). Two representative repeats of each sequence were chosen and considered as individual sequences for the multiple sequence alignment (Additional file 3).

Two WebLogos were created with these alignments. The first one, showed in Figure 3C, was done using the repeats of all sequences. The second one was done in order to create a WebLogo for consensus sequence (Figure 3D), and only those repeats of 25 and 26 amino acids were used in the alignment.

TSL was made to remark on the differences between the most abundant STs: type I and type IIa. Four different input alignments were made to create two TSL: 25 amino acid ST type I repeats versus 25 amino acid ST type IIa repeats and 26 amino acid ST type I repeats versus 26 amino acid ST type IIa repeats.

### Comparison between STs and cell wall proteins

The search for non-enzymatic cell wall proteins, namely AGPs, PRPs and HRGPs, was conducted firstly in the TAIR database ( http://www.arabidopsis.org/index.jsp), where *Arabidopsis thaliana* proteins are included. The Arabidopsis proteins were used to find cell wall proteins from other plants in the UniProt Knowledgebase ( http://www.ebi.ac.uk/Tools/sss/fasta/) using the FASTA program [79] at the EMBL-EBI web pages. The accession numbers of UniProtKB were used.

Alignments were done using the MegAlign program for multiple sequence alignment with the CLUSTAL W algorithm, using a bootstrap analysis of n=2000 for phylogenetic tree construction.

## Abbreviations

AGPs: Arabinogalactan proteins; DUF Domain: Domain of unknown function; EPR: Eicosapentapeptide repeat; EST: Expressed sequence tags; GRPs: Glycine-rich proteins; HRGPs: Hydroxyproline-rich glycoproteins; IUPs: Intrinsically unstructured proteins; LRR: Leucine-rich repeat; PPR: Pentatricopeptide repeat; PRPs: Proline-rich proteins; ST: ShooT specific protein; TPR: Tetratricopeptide repeat.

## Competing interests

The authors declare that they have no competing interests.

## Authors’ contributions

LA, IM and BD carried out the sequence searches, the sequence alignment and analysis of the sequences. RI and TJ carried out the study of the comparison of ST proteins versus cell wall proteins. BD and EL conceived of the study, and participated in its design and coordination and drafted the manuscript. All authors read and approved the final manuscript.

## Supplementary Material

Additional file 1 **Accession numbers of the sequences encoding proteins similar to ST proteins.** Sequences were obtained after different searches in EMBL databases as indicated in Methods. Full-length ORFs of chickpea *CarST1* and *CarST2* nucleotides sequences were used as well as the nucleotide sequences of their conserved repeats. Click here for file

Additional file 2 **Alphabetical list of plant species were sequences encoding ST proteins were found.** The file indicates the number of the different ST sequences found in each species and their names. Click here for file

Additional file 3 **ST deduced amino acid sequences organized by repeats and sorted by alphabetical order of the plant species.** The signal peptide is highlighted in grey; the first repeat is highlighted in green (except when it was chosen for the alignment); in blue, the repeats used for the analysis of alignment; in yellow, the Tyr in the repeats; in dark green, the putative phase shift. Red letters indicated Tyr, Trp and Cys in the mature N-terminal end before the repeats; pink letters indicated undefined amino acids. The putative N-glycosilation sites appeared underlined. The number of amino acids in the repeats refers to the most frequent one. Click here for file
