# Supplementary material for: ST proteins, a new family of plant tandem repeat proteins with a DUF2775 domain mainly found in Fabaceae and Asteraceae
Source: BMC Plant Biol. 2012 Nov 7;12:207. doi: 10.1186/1471-2229-12-207 (PMC3499167; doi:10.1186/1471-2229-12-207)
Supplement: Additional file 1 — Accession numbers of the sequences encoding proteins similar to ST proteins. Sequences were obtained after different searches in EMBL databases as indicated in Methods. Full-length ORFs of chickpea CarST1 and CarST2 nucleotides sequences were used as well as the nucleotide sequences of their conserved repeats. [file 1471-2229-12-207-S1.doc]

Additional file 1. **Accession numbers of the sequences encoding proteins similar to ST proteins.** Sequences were obtained after different searches in EMBL databases as indicated in Methods. Full-length ORFs of chickpea *CarST1* and *CarST2* nucleotides sequences were used as well as the nucleotide sequences of their conserved repeats. “Multiple sources” indicates that the mRNA to construct the library was obtained from a mixture of different organs and/or developmental stages and/or different growth conditions. Numbers indicate the order in which the sequences were found.

| **Specie** | # | **Accession #** | **Source** | **cDNA library** | **Name** |
| --- | --- | --- | --- | --- | --- |
| *Pisum sativum* | 1 | X51595 | gDNA | Not determined | PsaST2 |
|  | 2 | M16835 | mRNA | No information available | PsaST2 |
|  | 3 | X51594 | gDNA | Not determined | PsaST3 |
|  | 157 | JI907173 | mRNA | Leaf | PsaST1 |
|  | 158 | JI903736 | mRNA | Leaf | PsaST2 |
|  | 159 | JI912250 | mRNA | Leaf | PsaST2 |
|  | 160 | JI919869 | mRNA | Leaf | PsaST5 |
|  | 161 | JI897231 | mRNA | Leaf | PsaST4 |
|  | 162 | JI905697 | mRNA | Leaf | PsaST3 |
| *Striga asiatica* | 4 | DQ442389 | mRNA | Haustorium formation | SasST2 |
| *S. hermonthica* | 163 | FS498704 | mRNA | Strigol and exudates treated seedlings | SheST2 |
| *Medicago truncatula* | 5 | BG585224 | mRNA | *Glomus versiforme* inoculated root | MtrST1 |
|  | 6 | BG585526 | mRNA | *G. versiforme* inoculated root | MtrST1 |
|  | 7 | BG586160 | mRNA | *G. versiforme* inoculated root | MtrST1 |
|  | 8 | CB892521 | mRNA | *Sinorrhizobium meliloti* inoculated seedling root | MtrST1 |
|  | 9 | AW774592 | mRNA | *S. meliloti* inoculated seedling root | MtrST1 |
|  | 10 | AW585373 | mRNA | *G. versiforme* inoculated root | MtrST1 |
|  | 11 | AW171765 | mRNA | Pi starved root | MtrST1 |
|  | 38 | CA858247 | mRNA | Immature seed | MtrST3 |
|  | 39 | BE204687 | mRNA | Seedling root | MtrST3 |
|  | 40 | AA660785 | mRNA | Seedling root hair and root tips | MtrST3 |
|  | 41 | BE320352 | mRNA | Root in N_2_ limitation | MtrST4 |
|  | 42 | AW329068 | mRNA | Pi starved root | MtrST4 |
|  | 43 | AW329615 | mRNA | Pi starved root | MtrST4 |
|  | 44 | CA921263 | mRNA | Multiple sources | MtrST3 |
|  | 45 | CA920872 | mRNA | Multiple sources | MtrST3 |
|  | 46 | AW267799 | mRNA | *Phytophtora medicaginis* infected root | MtrST2 |
|  | 47 | CF068396 | mRNA | Multiple sources | MtrST2 |
|  | 48 | AW560950 | mRNA | *P. medicaginis* infected root | MtrST2 |
|  | 49 | AW329527 | mRNA | Pi starved root | MtrST2 |
|  | 50 | BM814423 | mRNA | *Meloidogyne incognita* infected root | MtrST2 |
|  | 51 | AW559473 | mRNA | *P. medicaginis* infected root | MtrST2 |
| **Specie** | # | **Accession #** | **Source** | **cDNA library** | **Name** |
| *M. truncatula* | 52 | BG448810 | mRNA | *Spodoptera exigua* local and systemic leaf | MtrST4 |
|  | 53 | BI265634 | mRNA | *S. exigua* local and systemic leaf | MtrST4 |
|  | 54 | BQ151571 | mRNA | Leaf | MtrST2 |
|  | 55 | AW559470 | mRNA | *P. medicaginis* infected root | MtrST2 |
|  | 56 | AW560833 | mRNA | *P. medicaginis* infected root | MtrST2 |
|  | 57 | AW267835 | mRNA | *P. medicaginis* infected root | MtrST2 |
|  | 58 | BM814686 | mRNA | *M. incognita* infected root | MtrST2 |
|  | 59 | BM814698 | mRNA | *M. incognita* infected root | MtrST2 |
|  | 60 | AW686310 | mRNA | *Rhizobium meliloti* inoculated root | MtrST4 |
|  | 61 | AJ504219 | mRNA | *Glomus intraradices* inoculated root | MtrST2 |
|  | 62 | BF004142 | mRNA | *R. meliloti* inoculated root | MtrST3 |
|  | 63 | BM814649 | mRNA | *M. incognita* infected root | MtrST2 |
|  | 64 | AW686950 | mRNA | Root in N_2_ limitation | MtrST2 |
|  | 65 | BE239373 | mRNA | Pi starved root | MtrST2 |
|  | 66 | AW560367 | mRNA | *P. medicaginis* infected root | MtrST4 |
|  | 67 | BM814860 | mRNA | *M. incognita* infected root | MtrST2 |
|  | 68 | AJ499880 | mRNA | *G. intraradices* inoculated root | MtrST6 |
|  | 69 | BE320482 | mRNA | Root in N_2_ limitation | MtrST3 |
|  | 70 | AJ500593 | mRNA | *G. intraradices* inoculated root | MtrST6 |
|  | 77 | AJ499913 | mRNA | *G. intraradices* inoculated root | MtrST6 |
|  | 78 | AW584777 | mRNA | *G. versiforme* inoculated root | MtrST6 |
|  | 79 | AJ500585 | mRNA | *G. intraradices* inoculated root | MtrST6 |
|  | 80 | AW586770 | mRNA | *G. versiforme* inoculated root | MtrST6 |
|  | 81 | AJ499401 | mRNA | *G. intraradices* inoculated root | MtrST6 |
|  | 82 | AJ500228 | mRNA | *G. intraradices* inoculated root | MtrST6 |
|  | 83 | AL386972 | mRNA | *G. intraradices* inoculated root | MtrST6 |
|  | 84 | AJ499500 | mRNA | *G. intraradices* inoculated root | MtrST6 |
|  | 85 | BG586163 | mRNA | *G. versiforme* inoculated root | MtrST6 |
|  | 86 | AJ499480 | mRNA | *G. intraradices* inoculated root | MtrST6 |
|  | 87 | AJ499826 | mRNA | *G. intraradices* inoculated root | MtrST6 |
|  | 88 | AJ500166 | mRNA | *G. intraradices* inoculated root | MtrST6 |
|  | 89 | AJ499985 | mRNA | *G. intraradices* inoculated root | MtrST6 |
|  | 90 | AL383432 | mRNA | *G. intraradices* inoculated root | MtrST6 |
|  | 147 | CR311718 | gDNA | Not determined | MtrST1 |
|  | 148 | CR489990 | gDNA | Not determined | MtrST2 |
|  | 150 | CR940310 | gDNA | Not determined | MtrST3 |
|  | 154 | AC146744 | gDNA | Not determined | MtrST4 |
|  | 155 | AC146744 | gDNA | Not determined | MtrST5 |
|  | 156 | CU468247 | gDNA | Not determined | MtrST6 |

| **Specie** | # | **Accession #** | **Source** | **cDNA library** | **Name** |
| --- | --- | --- | --- | --- | --- |
| *Helianthus argophyllus* | 13 | EE615920 | mRNA | Multiple sources | HarST1 |
|  | 164 | CF092113 | mRNA | Drought stressed multiple sources | HarST2 |
|  | 165 | EL514593 | mRNA | Multiple sources | HarST2 |
| *H. tuberosus* | 14 | EL467608 | mRNA | Multiple sources | HtuST1 |
|  | 126 | EL470284 | mRNA | Multiple sources | HtuST2 |
| *H. ciliaris* | 123 | EL425226 | mRNA | Multiple sources | HciST3 |
|  | 124 | EL423099 | mRNA | Multiple sources | HciST3 |
|  | 125 | EL423047 | mRNA | Multiple sources | HciST3 |
|  | 166 | EL427740 | mRNA | Multiple sources | HciST1 |
|  | 167 | EL425030 | mRNA | Multiple sources | HciST2 |
| *H. petiolaris* | 168 | DY948846 | mRNA | Multiple sources | HpeST2 |
| *H. paradoxus* | 169 | EL483694 | mRNA | Multiple sources | HpaST1 |
|  | 170 | EL477110 | mRNA | Multiple sources | HpaST2 |
| *Lactuca virosa* | 12 | DW172128 | mRNA | Multiple sources | LviST2 |
| *L. sativa* | 15 | DW137684 | mRNA | Multiple sources | LsaST2 |
|  | 16 | DW144449 | mRNA | Multiple sources | LsaST2 |
|  | 17 | DY984844 | mRNA | Multiple sources | LsaST2 |
|  | 18 | DW132067 | mRNA | Multiple sources | LsaST2 |
|  | 19 | DW132043 | mRNA | Multiple sources | LsaST2 |
|  | 20 | DW127400 | mRNA | Multiple sources | LsaST2 |
|  | 21 | DY980474 | mRNA | Multiple sources | LsaST2 |
|  | 22 | DW126565 | mRNA | Multiple sources | LsaST2 |
|  | 23 | DW123816 | mRNA | Multiple sources | LsaST2 |
|  | 24 | DW136748 | mRNA | Multiple sources | LsaST2 |
| *L. serriola* | 37 | DW106792 | mRNA | Multiple sources | LseST2 |
| *Phaseolus vulgaris* | 25 | CV543340 | mRNA | Pi starved root | PvuST1 |
|  | 171 | HS104538 | mRNA | Pi high root | PvuST1 |
| *Glycine max* | 26 | CF808904 | mRNA | *Phytophtora sojae* infected hypocotyl | GmaST1 |
|  | 27 | CF807005 | mRNA | *P. sojae* infected hypocotyl | GmaST1 |
|  | 28 | BE352736 | mRNA | Etiolated hypocotyl | GmaST1 |
|  | 29 | BE352692 | mRNA | Etiolated hypocotyl | GmaST1 |
|  | 30 | BE022154 | mRNA | *Bradyrhizobium japonicus* infected root | GmaST1 |
|  | 31 | BE347334 | mRNA | Hypocotyls and plumule | GmaST1 |
|  | 32 | AI461218 | mRNA | Root | GmaST1 |
|  | 33 | BF009715 | mRNA | hypocotyl | GmaST1 |
|  | 34 | BF009797 | mRNA | hypocotyl | GmaST1 |
|  | 35 | BI497520 | mRNA | *B. japonicus* infected root | GmaST1 |
|  | 71 | BF010300 | mRNA | Seedling epicotyl | GmaST1 |

| **Specie** | # | **Accession #** | **Source** | **cDNA library** | **Name** |
| --- | --- | --- | --- | --- | --- |
| *G. max* | 72 | CF920666 | mRNA | Root hair | GmaST1 |
|  | 73 | CX703688 | mRNA | Drought stressed root | GmaST1 |
|  | 74 | FK020973 | mRNA | Root with/without several stresses | GmaST1 |
|  | 75 | BI699502 | mRNA | Root | GmaST1 |
|  | 112 | BE352677 | mRNA | Etiolated hypocotyl | GmaST2 |
|  | 113 | BE554817 | mRNA | Etiolated hypocotyl | GmaST2 |
|  | 114 | AB266569 | mRNA | Differentially expressed in root after *Heterodera glycine* infection | GmaST2 |
|  | 115 | BG156156 | mRNA | Root | GmaST2 |
| *Senecio chrysanthemifolius* | 36 | DY662342 | mRNA | Immature capitulum bud | SchST1 |
| *S. aethnensis* | 172 | DY663305 | mRNA | Immature capitulum bud | SaeST1 |
| *Arachis duranensis* | 76 | GW938912 | mRNA | Root | AduST2 |
|  | 173 | GW938039 | mRNA | Root | AduST2 |
| *A. hypogaea* | 91 | EG530028 | mRNA | Seed | AhyST2 |
|  | 92 | EG530021 | mRNA | Seed | AhyST2 |
|  | 93 | EE124387 | mRNA | Seed | AhyST2 |
|  | 94 | EG530535 | mRNA | Seed | AhyST2 |
|  | 174 | GO323663 | mRNA | Developing embryo | AhyST2 |
| *A. stenosperma* | 95 | EH045555 | mRNA | *Meloidogyne arenaria* inoculated root | AstST2 |
|  | 96 | EH044565 | mRNA | *M. arenaria* inoculated root | AstST2 |
|  | 97 | EH047086 | mRNA | *M. arenaria* inoculated root | AstST2 |
|  | 98 | EH046834 | mRNA | *M. arenaria* inoculated root | AstST2 |
|  | 99 | EH045812 | mRNA | *M. arenaria* inoculated root | AstST2 |
| *Trifolium pratense* | 100 | BB908542 | mRNA | Three weeks old plant | TprST2 |
|  | 101 | BB905549 | mRNA | Three weeks old plant | TprST2 |
|  | 102 | BB914231 | mRNA | Three weeks old plant | TprST2 |
|  | 103 | BB912737 | mRNA | Three weeks old plant | TprST3 |
|  | 104 | BB926551 | mRNA | Leaf | TprST3 |
|  | 105 | BB902511 | mRNA | Three weeks old plant | TprST3 |
|  | 106 | BB918131 | mRNA | Leaf | TprST3 |
|  | 107 | BB904621 | mRNA | Three weeks old plant | TprST3 |
|  | 108 | BB912015 | mRNA | Three weeks old plant | TprST3 |
|  | 175 | HR241330 | gDNA | Not determined | TprST2 |
| *Lotus japonicus* | 109 | CB827406 | mRNA | *Mesorhizobium loti* induced nodule | LjaST1 |
|  | 110 | AW163929 | mRNA | *Mesorhizobium loti* induced root | LjaST1 |
|  | 111 | BE122541 | mRNA | *Mesorhizobium loti* induced root | LjaST1 |
|  | 146 | AP006373 | gDNA | Not determined | LjaST2 |
|  | 176 | FS338699 | mRNA | Multiple sources | LjaST1 |
| *Robinia pseudoacacia* | 116 | BI678066 | mRNA | Cambium and bark region | RpsST1 |

| **Specie** | # | **Accession #** | **Source** | **cDNA library** | **Name** |
| --- | --- | --- | --- | --- | --- |
| *Codonopsis lanceolata* | 117 | CF924254 | mRNA | Root | ClaST2 |
| *Betula pendula* | 118 | CD271649 | mRNA | *Paxillus involutus* inoculated root | BpeST2 |
|  | 119 | CD278517 | mRNA | Root | BpeST2 |
|  | 120 | CD269276 | mRNA | *P. involutus* inoculated root | BpeST2 |
|  | 121 | CD278107 | mRNA | Root | BpeST2 |
|  | 122 | CD277153 | mRNA | Root | BpeST2 |
| *Taraxacum kok-saghyz* | 127 | DR402388 | mRNA | Root | TkoST1 |
|  | 177 | GO670152 | mRNA | Root | TkoST1 |
| *T. officinale* | 178 | DY839229 | mRNA | Multiple sources | TofST1 |
|  | 179 | DY803365 | mRNA | Multiple sources | TofST2 |
| *Carthamus tinctorius* | 128 | EL375387 | mRNA | Multiple sources | CtiST2 |
|  | 180 | EL384565 | mRNA | Multiple sources | CtiST2 |
| *Centaurea maculosa* | 129 | EH711313 | mRNA | Multiple sources | CmaST2 |
|  | 130 | EH736685 | mRNA | Multiple sources | CmaST3 |
|  | 181 | EH743695 | mRNA | Multiple sources | CmaST3 |
| *Cyamopsis tetragonoloba* | 131 | EG976690 | mRNA | Seed | CteST1 |
|  | 132 | EG977303 | mRNA | Seed | CteST1 |
|  | 133 | EG982098 | mRNA | Seed | CteST1 |
|  | 134 | EG975456 | mRNA | Seed | CteST1 |
|  | 135 | EG978775 | mRNA | Seed | CteST1 |
|  | 136 | EG984259 | mRNA | Seed | CteST1 |
|  | 137 | EG979782 | mRNA | Seed | CteST1 |
|  | 138 | EG987204 | mRNA | Seed | CteST1 |
|  | 139 | EG978698 | mRNA | Seed | CteST1 |
|  | 140 | EG982531 | mRNA | Seed | CteST1 |
|  | 141 | EG980908 | mRNA | Seed | CteST1 |
|  | 142 | EG986094 | mRNA | Seed | CteST1 |
|  | 143 | EG980663 | mRNA | Seed | CteST1 |
|  | 144 | EG983298 | mRNA | Seed | CteST1 |
|  | 145 | EG980617 | mRNA | Seed | CteST1 |
| *Vitis amurensis* | 182 | GW665914 | mRNA | Pericarp | VamST2 |
| *V. vinifera* | 149 | AM438648 | gDNA | Not determined | VviST2 |
|  | 151 | AM455744 | gDNA | Not determined | VviST5 |
|  | 152 | AM426829 | gDNA | Not determined | VviST4 |
|  | 153 | AM486941 | gDNA | Not determined | VviST6 |
|  | 183 | EC949568 | mRNA | Flower, leaf and root | VviST3 |
|  | 184 | CF604673 | mRNA | Root | VviST3 |
|  | 185 | CX126879 | mRNA | Fruit | VviST2 |

## .

| **Specie** | # | **Accession #** | **Source** | **cDNA library** | **Name** |
| --- | --- | --- | --- | --- | --- |
| *V. vinifera* | 186 | EE084826 | mRNA | GA_3_ treated fruit | VviST2 |
|  | 187 | EE066803 | mRNA | Bud | VviST2 |
| *Cajanus cajan* | 188 | GR471603 | mRNA | Leaf challenged by sterility mosaic disease | CcaST2 |
| *Cicer arietinum* | 189 | X97454 | mRNA | Etiolated 5-d-old epicotyl | CarST1 |
|  | 190 | X97455 | mRNA | Etiolated 5-d-old epicotyl | CarST2 |
|  | 191 | GR409003 | mRNA | Field drought stressed root | CarST1 |
|  | 192 | GR409056 | mRNA | Field drought stressed root | CarST1 |
|  | 193 | GR398946 | mRNA | Field drought stressed root | CarST1 |
|  | 194 | GR394887 | mRNA | Field drought stressed root | CarST1 |
|  | 195 | GR396384 | mRNA | Field drought stressed root | CarST1 |
|  | 196 | GR392185 | mRNA | Field drought stressed root | CarST1 |
|  | 197 | GR397510 | mRNA | Field drought stressed root | CarST1 |
|  | 198 | GR397964 | mRNA | Field drought stressed root | CarST1 |
|  | 199 | GR399070 | mRNA | Field drought stressed root | CarST1 |
|  | 200 | GR397104 | mRNA | Field drought stressed root | CarST2 |
|  | 201 | GR397054 | mRNA | Field drought stressed root | CarST2 |
|  | 202 | GR391723 | mRNA | Field drought stressed root | CarST2 |
|  | 203 | GR405462 | mRNA | Salinity stressed root | CarST1 |
|  | 204 | GR405463 | mRNA | Salinity stressed root | CarST1 |
|  | 205 | GR403368 | mRNA | Salinity stressed root | CarST1 |
|  | 206 | GR405543 | mRNA | Salinity stressed root | CarST1 |
|  | 207 | GR408596 | mRNA | Salinity stressed root | CarST1 |
|  | 208 | GR401697 | mRNA | Salinity stressed root | CarST1 |
|  | 209 | GR420995 | mRNA | Salinity stressed root | CarST1 |
|  | 210 | GR408828 | mRNA | Salinity stressed root | CarST2 |
|  | 211 | GR405394 | mRNA | Salinity stressed root | CarST2 |
|  | 212 | GR408027 | mRNA | Salinity stressed root | CarST2 |
|  | 213 | GR407317 | mRNA | Salinity stressed root | CarST2 |
|  | 214 | GR407673 | mRNA | Salinity stressed root | CarST2 |
|  | 215 | GR406833 | mRNA | Salinity stressed root | CarST2 |
|  | 216 | GR408735 | mRNA | Salinity stressed root | CarST2 |
|  | 217 | GR403640 | mRNA | Salinity stressed root | CarST2 |
|  | 218 | GR402381 | mRNA | Salinity stressed root | CarST2 |
|  | 219 | GR406783 | mRNA | Salinity stressed root | CarST2 |
|  | 220 | GR408590 | mRNA | Salinity stressed root | CarST2 |
|  | 221 | GR400291 | mRNA | Slow drough root | CarST2 |
|  | 222 | DY475186 | mRNA | Stem and leaf after *Ascochyta rabiei* inoculation | CarST2 |

| **Specie** | # | **Accession #** | **Source** | **cDNA library** | **Name** |
| --- | --- | --- | --- | --- | --- |
| *C. arietinum* | 223 | FL512343 | mRNA | Drought-stressed root | CarST1 |
|  | 224 | GR915176 | mRNA | *Fusarium oxysporum* infected root and collar | CarST1 |
|  | 225 | GR915260 | mRNA | *F. oxysporum* infected root and collar | CarST1 |
|  | 226 | GR917690 | mRNA | *F. oxysporum* infected root and collar | CarST1 |
|  | 227 | GR915965 | mRNA | *F. oxysporum* infected root and collar | CarST1 |
| *Glycyrrhiza uralensis* | 228 | FS266740 | mRNA | Rhizome | GurST2 |
| *Lens culinaris* | 229 | JI847938 | mRNA | No information available | LcuST1 |
| *Lupinus albus* | 230 | FG090480 | mRNA | Root | LalST2 |
|  | 231 | FF837384 | mRNA | Phosphate stressed root | LalST1 |
| *Oxytropis campestris + O. splendens* | 232 | GW698114 | mRNA | Seedling mixture of both | OcsST2 |
|  | 233 | GW698076 | mRNA | Seedling mixture of both | OcsST3 |
| *Vigna unguiculata* | 234 | FF398053 | mRNA | Root | VunST1 |
|  | 235 | FG895280 | mRNA | Multiple sources | VunST1 |
|  | 236 | FF391220 | mRNA | Root | VunST2 |
|  | 237 | FF388191 | mRNA | Root | VunST2 |
|  | 238 | FC458206 | mRNA | Multiple sources | VunST2 |
|  | 239 | FF401342 | mRNA | Root | VunST2 |
|  | 240 | FF540053 | mRNA | Leaf and shoot | VunST1 |
|  | 241 | FC458205 | mRNA | Multiple sources | VunST1 |
| *Artemisia annua* | 242 | EZ180889 | mRNA | Cotyledon | AanST2 |
| *Barnadesia spinosa* | 243 | GE546541 | mRNA | Multiple sources | BspST2 |
| *Cichorium endivia* | 244 | EL370672 | mRNA | Multiple sources | CenST1 |
| *Parthenium argentatum* | 245 | GW782895 | mRNA | Bark in cold acclimated plant | ParST1 |
| *Saussurea medusa* | 246 | FF147576 | mRNA | Callus | SmeST2 |
| *Catharanthus roseus* | 247 | FD422994 | mRNA | Root | CroST2 |
|  | 248 | FD425188 | mRNA | Information not available | CroST3 |
| *Salvia miltiorrhiza* | 249 | CV172190 | mRNA | Whole youth plant | SmiST2 |
| *Coffea arabica* | 250 | GT679861 | mRNA | BTH treated hypocotyls | CoaST2 |
|  | 251 | GW478754 | mRNA | Mature leaves | CoaST2 |
|  | 252 | GW473053 | mRNA | Floral buds | CoaST2 |
| *Sesamum indicum* | 253 | BU670525 | mRNA | Developing seed | SinST2 |
| *Anthirrhinum majus* | 254 | AJ808567 | mRNA | Multiple sources | AmaST1 |
|  | 255 | AJ789720 | mRNA | Multiple sources | AmaST2 |
| *Mimulus guttatus* | 256 | GR173712 | mRNA | Multiple sources | MguST2 |
|  | 257 | GR131953 | mRNA | Floral buds | MguST2 |
| *Triphysaria versicolor* | 258 | DR175605 | mRNA | Root tip in early haustorium | TveST2 |
|  | 259 | EX987395 | mRNA | Root tip | TveST2 |
|  | 260 | EX987546 | mRNA | Root tip | TveST2 |

| **Specie** | # | **Accession #** | **Source** | **cDNA library** | **Name** |
| --- | --- | --- | --- | --- | --- |
| *Capsicum annuum* | 261 | CA520654 | mRNA | No information available | CanST2 |
| *Nicotiana benthamiana* | 262 | EX533939 | mRNA | Isolated stem trichome | NbeST1 |
| *N. sylvestris* | 263 | BP751999 | mRNA | Root | NsyST1 |
| *N. tabacum* | 264 | FH154273 | gDNA | Not determined | NtaST2 |
|  | 265 | EB441543 | mRNA | Leaf | NtaST2 |
|  | 266 | FG640012 | mRNA | Leaf trichome | NtaST2 |
| *Petunia axillaris* | 267 | FN009560 | mRNA | Corolle tubes | PaxST1 |
| *Solanum lycopersicum* | 268 | ES896127 | mRNA | Isolated trichome | SliST1 |
| *S. melongena* | 269 | FS053279 | mRNA | Root | SomST1 |
|  | 270 | FS086922 | mRNA | Multiple sources | SomST2 |
| *S. nigrum* | 271 | GU594271 | mRNA | No information available | SniST2 |
| *S. tuberosum* | 272 | BQ513154 | mRNA | Multiple sources | StuST3 |
|  | 273 | JG565498 | mRNA | Root fertilised with N_2_ | StuST3 |
|  | 274 | JG564809 | mRNA | Root fertilised with N_2_ | StuST2 |
|  | 275 | JG700022 | mRNA | Epicotyl of enlarging tuber | StuST2 |
|  | 276 | DN922887 | mRNA | *Streptomyces scabei* challenged tuber | StuST2 |
| *Panax ginseng* | 277 | DV554014 | mRNA | Leaf | PgiST2 |
|  | 278 | JI158499 | mRNA | Root | PgiST3 |
| *P. quinquefolius* | 279 | GR873237 | mRNA | Root | PquST2 |
| *Jatropha curcas* | 280 | EZ418831 | mRNA | Seed | JcuST2 |
| *Ricinus comunis* | 281 | EE256043 | mRNA | Leaf | RcoST2 |
|  | 282 | EQ973834 | gDNA | Not determined | RcoST3 |
| *Eucalyptus globulus* | 283 | GW337769 | mRNA | Root apex | EglST2 |
| *Ribes nigrum* | 284 | GT027593 | mRNA | Leaf bud | RniST2 |
| *Fragaria x ananassa* | 285 | CO817331 | mRNA | Salicylic acid treated whole plant | FanST2 |
| *Prunus armeniaca* | 286 | CB822249 | mRNA | Fruit | PraST1 |
| *P. persica* | 287 | ES216467 | mRNA | Mostly cambium | PpeST2 |
|  | 288 | GE653325 | mRNA | Apical bud tips | PpeST2 |
|  | 289 | FE969363 | mRNA | Mostly cambium | PpeST2 |
| *Rosa hybrida* | 290 | EC586680 | mRNA | Apex | RhiST2 |
| *Citrus jambhiri* | 291 | CO912681 | mRNA | Root | CjaST2 |
| *C. paradisi x C. trifoliata* | 292 | CX670876 | mRNA | Nematode challenged root | CptST2 |
| *C. reticulata* | 293 | EY760765 | mRNA | Leaf | CreST2 |
| *C. sinensis* | 294 | CK935556 | mRNA | Developing fruit | CsiST2 |
|  | 295 | BQ622984 | mRNA | Entire seedling | CsiST2 |
| *Casuarina glauca* | 296 | FQ371558 | mRNA | Non inoculated root | CglST2 |
|  | 297 | FQ318367 | mRNA | *Frankia* inoculated root | CglST2 |

| **Specie** | # | **Accession #** | **Source** | **cDNA library** | **Name** |
| --- | --- | --- | --- | --- | --- |
| *Alnus glutinosa* | 298 | FQ354931 | mRNA | Root | AglST2 |
|  | 299 | FQ348081 | mRNA | Root | AglST2 |
|  | 300 | FQ349891 | mRNA | Root | AglST2 |
|  | 301 | FQ338239 | mRNA | *Frankia alni* inoculated root | AglST2 |
| *Castanea mollissima* | 302 | GO920850 | mRNA | Root | CmoST2 |
| *Q. petraea* | 303 | FN765663 | mRNA | Root | QpeST1 |
|  | 304 | FN769833 | mRNA | Root | QpeST1 |
|  | 305 | FK857934 | mRNA | *Lactarius quietus* ectomycorrhiza/root | QpeST1 |
|  | 306 | FN766467 | mRNA | Root | QpeST1 |
| *Quercus robur* | 307 | CU639636 | mRNA | Hypoxia stressed root | QroST1 |
|  | 308 | FN999225 | mRNA | Green leaves | QroST1 |
|  | 309 | FN739047 | mRNA | Root | QroST1 |
| *Juglans hindsii x J. regia* | 310 | EL898822 | mRNA | *Pratylenchus vulnus* infected root | JhrST2 |
|  | 311 | EL901111 | mRNA | Root | JhrST3 |
| *Gossypium hirsutum* | 312 | CD486530 | mRNA | Root and hypocotyl | GhiST2 |
|  | 313 | FG551651 | mRNA | Embryonic callus | GhiST2 |
|  | 314 | ES811724 | mRNA | Fiber | GhiST2 |
| *Theobroma cacao* | 315 | CU575341 | mRNA | Hypocotyl | TcaST2 |
| *Corchorus capsularis* | 316 | FK826446 | mRNA | Stem | CocST3 |
|  | 317 | FK826550 | mRNA | Bast fiber and associated tissue | CocST2 |
| *Populus deltoides* | 318 | CX167518 | mRNA | *Marssonia* inoculated leaf | PdeST2 |
| *P. euphratica* | 319 | AJ779424 | mRNA | Root | PeuST2 |
| *P. trichocarpa* | 320 | EF148072 | mRNA | Leaf and green stem | PtrST3 |
|  | 321 | CN518873 | mRNA | Primary xylem and primary phloem | PtrST3 |
| *Actinidia arguta* | 322 | FG483557 | mRNA | Fruit | AarST2 |
| *A. chinensis* | 323 | FG458758 | mRNA | Fruit | AchST2 |
| *Citrullus lanatus* | 324 | GD179440 | mRNA | Fruit | CilST2 |
| *Cucumis melo* | 325 | AM729386 | mRNA | Root | CmeST2 |
| *C. sativus* | 326 | GS818240 | mRNA | Young leaves | CsaST1 |
| *Aquilegia formosa x A. pubescens* | 327 | DR929375 | mRNA | Multiple sources | AfpST2 |
