# Supplementary material for: ST proteins, a new family of plant tandem repeat proteins with a DUF2775 domain mainly found in Fabaceae and Asteraceae
Source: BMC Plant Biol. 2012 Nov 7;12:207. doi: 10.1186/1471-2229-12-207 (PMC3499167; doi:10.1186/1471-2229-12-207)
Supplement: Additional file 2 — Alphabetical list of plant species were sequences encoding ST proteins were found. The file indicates the number of the different ST sequences found in each species and their names. [file 1471-2229-12-207-S2.doc]

## Additional file 2. Alphabetical list of plant species were sequences encoding ST proteins were found. The number of the different ST sequences found in each species and their names are indicated.

| **Species** | # | **Name** |
| --- | --- | --- |
| *Actinidia arguta* | 1 | AarST2 |
| *A. chinensis* | 1 | AchST2 |
| *Alnus glutinosa* | 1 | AglST2 |
| *Anthirrhinum majus* | 2 | AmaST1, AmaST2 |
| *Aquilegia caerulea* | 1 | AcaST2 |
| *A. formosa x A. pubescens* | 1 | AfpST2 |
| *Arachis duranensis* | 1 | AduST2 |
| *A. hypogaea* | 1 | AhyST2 |
| *A. stenosperma* | 1 | AstST2 |
| *Artemisia annua* | 1 | AanST2 |
| *Barnadesia spinosa* | 1 | BspST2 |
| *Betula pendula* | 1 | BpeST2 |
| *Cajanus cajan* | 1 | CcaST2 |
| *Capsicum annuum* | 1 | CanST2 |
| *Carthamus tinctorius* | 1 | CtiST2 |
| *Castanea mollissima* | 1 | CmoST2 |
| *Casuarina glauca* | 1 | CglST2 |
| *Catharanthus roseus* | 2 | CroST2, CroST3 |
| *Centaurea maculosa* | 2 | CmaST2, CmaST3 |
| *Cicer arietinum* | 2 | CarST1, CarST2 |
| *Cichorium endivia* | 1 | CenST1 |
| *Citrullus lanatus* | 1 | CilST2 |
| *Citrus jambhiri* | 1 | CjaST2 |
| *C. paradisi x C. trifoliata* | 1 | CptST2 |
| *C. reticulata* | 1 | CreST2 |
| *C. sinensis* | 1 | CsiST2 |
| *Codonopsis lanceolata* | 1 | ClaST2 |
| *Coffea arabica* | 1 | CoaST2 |
| *Corchorus capsularis* | 2 | CocST2, CocST3 |
| *Cucumis melo* | 1 | CmeST2 |
| *C. sativus* | 2 | CsaST1, CsaST2 |
| *Cyamopsis tetragonoloba* | 1 | CteST1 |
| *Eucalyptus globulus* | 1 | EglST2 |
| *E. grandis* | 2 | EgrST2, EgrST3 |
| *Fragaria x ananassa* | 1 | FanST2 |

| **Species** | # | **Name** |
| --- | --- | --- |
| *Glycine max* | 2 | GmaST1, GmaST2 |
| *Glycyrrhiza uralensis* | 1 | GurST2 |
| *Gossypium hirsutum* | 1 | GhiST2 |
| *Helianthus argophyllus* | 2 | HarST1, HarST2 |
| *H. tuberosus* | 2 | HtuST1, HtuST2 |
| *H. ciliaris* | 3 | HciST2, HciST3, HciST4 |
| *H. petiolaris* | 1 | HpeST2 |
| *H. paradoxus* | 2 | HpaST1, HpaST2 |
| *Jatropha curcas* | 1 | JcuST2 |
| *Juglans hindsii x J. regia* | 2 | JhrST2, JhrST3 |
| *Lactuca sativa* | 1 | LsaST2 |
| *L. serriola* | 1 | LseST2 |
| *L. virosa* | 1 | LviST2 |
| *Lens culinaris* | 1 | LcuST1 |
| *Lotus japonicus* | 2 | LjaST1, LjaST2 |
| *Lupinus albus* | 2 | LalST1, LalST2 |
| *Manihot sculenta* | 4 | MesST2, MesST3, MesST4, MesST5 |
| *Medicago truncatula* | 6 | MtrST1, MtrST2, MtrST3, MtrST4, MtrST5, MtrST6 |
| *Mimulus guttatus* | 1 | MguST2 |
| *Nicotiana benthamiana* | 1 | NbeST1 |
| *N. sylvestris* | 1 | NsyST1 |
| *N. tabacum* | 1 | NtaST2 |
| *Oxytropis campestris + O. splendens* | 2 | OcsST2, OcsST3 |
| *Panax ginseng* | 2 | PgiST2, PgiST3 |
| *P. quinquefolius* | 1 | PquST2 |
| *Parthenium argentatum* | 1 | ParST1 |
| *Petunia axillaris* | 1 | PaxST1 |
| *Phaseolus vulgaris* | 1 | PvuST1 |
| *Pisum sativum* | 5 | PsaST1, PsaST2, PsaST3 PsaST4, PsaST5 |
| *Populus deltoides* | 1 | PdeST2 |
| *P. euphratica* | 1 | PeuST2 |
| *P. trichocarpa* | 2 | PtrST2, PtrST3 |
| *Prunus armeniaca* | 1 | PraST1 |
| *P. persica* | 2 | PpeST2, PpeST3 |
| *Quercus petrae* | 1 | QpeST1 |
| *Q. robur* | 1 | QroST1 |
| *Ribes nigrum* | 1 | RniST2 |
| *Ricinus comunis* | 2 | RcoST2, RcoST3 |
| *Robinia pseudoacacia* | 1 | RpsST1 |

| **Species** | # | **Name** |
| --- | --- | --- |
| *Rosa hybrida* | 1 | RhiST2 |
| *Salvia miltiorrhiza* | 1 | SmiST2 |
| *Saussurea medusa* | 1 | SmeST2 |
| *Senecio aethnensis* | 1 | SaeST1 |
| *S. chrysanthemifolius* | 1 | SchST1 |
| *Sesamum indicum* | 1 | SinST2 |
| *Solanum lycopersicum* | 1 | SliST1 |
| *S. melongena* | 2 | SomST1, SomST2 |
| *S. nigrum* | 1 | SniST2 |
| *S. tuberosum* | 2 | StuST2, StuST3 |
| *Striga asiatica* | 1 | SasST2 |
| *S. hermonthica* | 1 | SheST2 |
| *Taraxacum kok-saghyz* | 1 | TkoST1 |
| *T. officinale* | 2 | TofST1, TofST2 |
| *Theobroma cacao* | 1 | TcaST2 |
| *Trifolium pratense* | 2 | TprST2, TprST3 |
| *Triphysaria versicolor* | 1 | TveST2 |
| *Vigna unguiculata* | 2 | VunST1, VunST2 |
| *Vitis amurensis* | 1 | VamST2 |
| *Vitis vinifera* | 5 | VviST2, VviST3, VviST4, VviST5, VviST6 |
