# Supplementary material for: ST proteins, a new family of plant tandem repeat proteins with a DUF2775 domain mainly found in Fabaceae and Asteraceae
Source: BMC Plant Biol. 2012 Nov 7;12:207. doi: 10.1186/1471-2229-12-207 (PMC3499167; doi:10.1186/1471-2229-12-207)
Supplement: Additional file 3 — ST deduced amino acid sequences organized by repeats and sorted by alphabetical order of the plant species. The signal peptide is highlighted in grey; the first repeat is highlighted in green (except when it was chosen for the alignment); in blue, the repeats used for the analysis of alignment; in yellow, the Tyr in the repeats; in dark green, the putative phase shift. Red letters indicated Tyr, Trp and Cys in the mature N-terminal end before the repeats; pink letters indicated undefined amino acids. The putative N-glycosilation sites appeared underlined. The number of amino acids in the repeats refers to the most frequent one. [file 1471-2229-12-207-S3.doc]

**Additional file 3.** **ST deduced amino acid sequences organized by repeats and sorted by alphabetical order of the plant species.** The signal peptide is highlighted in grey; the first repeat is highlighted in green (except when it was chosen for the alignment); in blue, the repeats used for the analysis of alignment; in yellow, the Tyr in the repeats; in dark green, the putative phase shift. Red letters indicated Tyr, Trp and Cys in the mature N-terminal end before the repeats; pink letters indicated undefined amino acids. The putative N-glycosilation sites appeared underlined. The number of amino acids in the repeats refers to the most frequent one.

## *Actinidia arguta* ST2 (AarST2). C-terminal, 4 repeats, 25 amino acids. Subtype IIa

DFEPRPNVSAYGDDTKLTEEKSFVKDFEPRPNVSAYSDDTKLTGEKSFVKDFEPRPNISAYGDDAGLKDEKAFVKDFEPRPNVSVYQN

## *A. chinensis* ST2 (AchST2). Full length, 4 repeats, 25 amino acids. Subtype IIa

PSFAFFTLFSLVLLANAIDA

RPDPGEYWQGVMKDQPMPEAIQGLIHQGDSVSPVSNKKRDCHTSTNAKEKPFVK

DFDPRPNVSAYSDNTKLTGEKSFVKDFEPRPNLSAYSDDTKLTEEKSFVKDFEPRPNVSAYSDDAGLKDEKAFVKDFEPRPNVSVYQN

## *Alnus glutinosa* ST2 (AglST2). N-terminal, 5 repeats, 26 amino acids. Subtype IIa

MNPRMAWLTLLSLLLFSISVES

RKDPGEYWTSVMKDQPMPEAIQGLVHLDSAPSQPTKNANCDHSSMGTRNKNQPFAE

DFEPRPNVSAYNDDDVGQEKEKKFVKGFEPRPDVTAYNDDDVGGEKEKKFVKDFEPRPNVSAYNDDDVGQEKEKKFVKDFEPRPNVSAYNDDDVGREKEKKFVKDFEPRPNLSAYNDDDVGQETEKKFV

## *Anthirrhinum majus* ST1 (AmaST1). N-terminal, 6 repeats, 25 amino acids. Type I

MKSLLISVGLLCFALFAYDVEA

RKDPGEYWRDATKDNIMPKSVESSIDVAKVTSEDNDKNHCHTSTESSGSIPTSEHHKSYTK

DFEPRPSVTSYNNEINPKGGKLFTKDFEPRPSATGYSDDISPKSDKLFSKDFEPRPSASGYSDDVSPKGENLFANDFEPRPSITGYNNDIHPKGDKFFSKDFEPRPSATGYSNDINPKGENLFAXDFEPRPSVTGYKQ

## *A. majus* ST2 (AmaST2). Full length, 7 repeats, 25 amino acids. Subtype IIa

MKSLLISVGLLYFALFAYDVEA

RKDPGEYWRDATKDRMMPKSIEGSIDVAKVTSEENGTNHCHTSTESSSSILN

DFESRPSLTGYIDGVNPNGSKSLTKEFEQYTSFSSDGTKPKEIVSYTK

DFEPRPNLSSYNDNDELKDDNSFLRNFEPRPNLSSYNDNGELKDENSFVRNFEPRPNLSSYNDNGELKDENSLVRNFKPRPNSVAINNDDAIRAEAQK

DFEPRPNLSVYHE

## Aquilegia caerulea ST2 (AcaST2). Full length, 2 repeats, 25 amino acids. Subtype IIa

MLHYYFASIINA

RTAPEEYWKQVMKDQPMPDALKGLVDSNPSESVSTVMIKTDCHTSLNTQDNKHLANDILADPGVTVLYPDEDEKSFVM

DFEPRPNLSVYHDGELKGQKKAFAEDFEPRPNVSVYHD

## *A. formosa x A. pubescens* ST2 (AfpST2). Full length, 3 repeats, varied amino acids. Subtype IIa

MESLYGFFALFSLLLFVTTINA

RTAPEEYWIQVMKDQPMPEALKGLVNSNPSESVSKDKIKTDCHTSLNTQENKHLPNDVLADPGSLTAFYGDVKPKENEDTIDNKHLAMNFLADASITVFYGHAATENEEKAFVK

DFEPRPNVSVYHDEEDKSFVK

DFEPRPNVSVYHDGELKDQKKAFADDLEPRPNLSVYHD

## *Arachis duranensis* ST2 (AduST2). Full length, 4 repeats, 26 amino acids. Subtype IIa

MRVAFAFLPLAFLVLLVASVES

RKDAGEYWKMIKDMPEEIQGLVKFNEEQQTEGLKPLMVTEEVTMEKKVFTEDNEMDCKEKKQVVK

EFEPRPNVSAYGDNEVDAKEKKHVVNDFEPRPNVSAYGDNDVDAKNKKHVVNDFEPRPNVSAYGDNDIDAKKQKKFVKSFEPRPNVSVYDKDNVDAK

## *A. hypogea* ST2 (AhyST2). Full length, 4 repeats, 26 amino acids. Subtype IIa

MRVAFAFLPHAFLVLLVASVES

RKDAGEYWKMIKDMPEEIQGLVKFNEEQQIEGLKPLMVTEEVTMEKKVFTEDNEMDCKEKKQVVK

EFEPRPNVSAYGDNEVDAKGKKHVVNDFEPRPNVSAYGDNDVDAKNKKHVVNDFEPRPNVSAYGDNDINAKKQKKFVRSFEPRPNVSAYGKDNVDAK

## A. stenosperma ST2 (AstST2). Internal, 4 repeats, 26 amino acids. Subtype IIa

TKTNVSSYGDNEVDAKEKKHVVNDFEPRPNVSAYGDNDVDAKNKKHVVNDFEPRPNVSAYGDNDIDAKKQKKFVKSFEPRPNVSVYDKDNVDAK

## *Artemisia annua* ST2 (AanST2). Full length, 4 repeats, 25 amino acids. Subtype IIa

MESSLSFFALFSLIMIANVIDA

RPNPGEYKPNSLPTKKSHCLTNEKKFEQNK

DFEPRPNISVYEHSEGLKAEMFDK

DFEPRPNISTYDNGASLNSKKTSQEEFEPRPNISVYDNGVGLNGKKTLDGEFEPRPNISVYNG

## *Barnadesia spinosa* ST2 (BspST2). Full length, 5 repeats, 26 amino acids. Subtype IIa

MESSFAFLALLSLILVTTTVVA

RPDTGEYWKDSFVGRGTLVSSLPTKKNDCHTSTMPKDHNQVAKGKKFVK

DFEPKPNISAYHDDXKVTGDRKSFKEDSEQNPNVSVYNNDVIAKEEKKKIVKDFEPRPNVPVHDNGLEDEGKFDK

DFEPRPNVSAYDNNTGLKDKKTFEKDFEPRPNVSVYDD

## *Betula pendula* ST2 (BpeST2). Full length, 4 repeats, 26 amino acids. Subtype IIa

MMNPPISWLTLLSLLLFAISIES

GKDPGEYWTSVMKDQPMPEAIKGLVHLDSAPSQPTKNANCDHPSVETRNKNQHFAE

DFEPRPNISAYSDDDVGQKTEKNFVKDFEPRPNISSYNDDDVGKETQKKFVKDFEPRPNISSYNDDDVGQKFVK

DFEPRPDVTSYRE

## *Cajanus cajan* ST2 (CcaST2). C-terminal, 4 repeats, 26 amino acids. Subtype IIa

RGDNEIDFKEKKETLE

DFEPRPNISAYGDNEIDFKEKKEAFEDFEPRPNISTYGDNGIGSKKKEVVK

DFVSRPNISTYENNNIDDEINEKLKVDFESRPSATKYDA

## *Capsicum annuum* ST2 (CanST2). Internal, 2 repeats, 22 amino acids. Subtype IIa

MSQCPKAIQHLMPRSHKEIKTDCDKSS

DFEPIPNVSSYHDESKLKEEEKDFEPRPNVSSYHDGDAGPQQEK

## *Carthamus tinctorium* ST2 (CtiST2). N-terminal, 7 repeats, 25 amino acids. Subtype IIa

MKSPLPFLVLFSLIMITISIDA

RSIPKVHWQDSFVRRGTSVSSLATKKGLCHTLAETKGQRSSSNEQVLIHDYNVNQDSSNN

DFEPRPNISVYDDGAGLKGKKTVDEDFEPRPNISVYDNDVGLKGTKTSGEEFEPRPNISLYDNGVGLKGTKTSNEDFEPRPNISVYDNGVGLKATKTSNEDFEPRPNISVYDDGVGLKATKTSNEDFEPRPNISVYDNGVGLKGTKTSNEDFEPRPNISVYDNGVGLKGTKTSNEDFEPSQTSLFMTMVLV

## *Castanea mollissima* ST2 (CmoST2). Full length, 2 repeats, 26 amino acids. Type not determined

MIVDMESRFAWLTLFSLLLFANTTES

RKDPGEYWTNVTKDQPMPEAIQGLVHLDSSPSKLSKRDNCHTSEGATSKDQAYVNVVDPETKKKFVN

DFERRPNISACNDDDVGSKEEKPFVEDFEPRPSASVYSD

## *Casuarina glauca* ST2 (CglST2). N-terminal, 6 repeats, 26 amino acids. Type III

MCSRVAWLALFALLAFAMTMEA

RKDPGEYWATVMQDQPMPEAIQGLLQSDTTQSQPHKHSNCHSSDVTRNNHQLVQDSRHENGKPFAK

DFEPRPDISAYNDDDVGAQKQQKFVKDFKPRPDVSAYNDDDFGEQKQKNFVKDFKPRPDVTAYNDDDVGAQKQQKFVKDFKPQPDISAYNDDDFGEQKQKKFVKDFKPRPDVTAYNDDDVGAQKQQKFVKDFKPRADISAYNDDDLGEQ

## *Catharanthus roseus* ST2 (CroST2). Internal, 3 repeats, 26 amino acids. Subtype IIa

IQRIRHEVSSYPDDAKLKTDETRFKK

DFEPRPNVSTYPDKDDGLKTQKEFMKDFEPRPNVSVYDA

## *C. roseus* ST3 (CroST3). Internal, 6 repeats, 26 amino acids. Type not determined

GSKEFGTRAKLKEETTIFKR

DFEPIPSATSYPDDAKLKDEITTFKRDFEPRPSATSYPDDTKLKHETSTFKRDFEPRPSATSYPDETRFKK

DFEPRPNVSAYLDKDGLKTQKEFMKDFEPRPNLSAYPDKDGLKHKRIHE

DFEPRPNLSAYPD

## *Centaurea maculosa* ST2 (CmaST2). N-terminal, 8 repeats, 25 amino acids. Subtype IIa

MESSLAFLVLLSLILTAIMNIDA

RPHREEYLQDSFVRRGTSVSPQPIKKSHCNTFAKASNHISSGHD

DFEPRPNVSSYGNDVNPDGNKK

DFEPRPNVSVYDEDTSLKGKENFKGEFEPRPNLSVYDNNIGLKGKSKVDEEFEPRPNVSVYDNDTSLKGKKNANEEFEPRPNVSIYDNDTGLRSKKNVNEEFEPRPNLSVYDKDTGLKGKKNVDEAFEPRPNLSVYDNAAGLKGKKNVDEEFEPRPN

## *C. maculosa* ST3 (CmaST3). N-terminal, 7 repeats, 25 amino acids. Subtype IIa

MKSPLPCLALFSFIMITITIDA

RPIPKVDWQDSIVRRGTSVSPLATKKVHCHTLAETKGQSSSSNEQVLVHDYNVNQDSSNN

DFEPRPNISVYDDGASLKGKKTVDEEFEPRPNISVYDNDLGLKGTKTSGEEFEPRPNISVYDNDLGLKGTKTTGEEFEPRPNISVYDNDVGLKGTKSSNEDFEPRPNISVYDNGVGLKGTKSSNEDFEPRPNVSVYDNGVGLKGTNSSNEDFEPRPNVSVY

## *Cicer arietinum* ST1 (CarST1). Full length, 9 repeats, 25 amino acids. Type I

MRPAVAALIFLFLFLFAATIES

RNDLKEYWKTIMKDEEMPVGIEGLLQLKPEIEPVKNYKIKEEHAKGKCDQHPITNTLNIIEKKVITEEFESKPTISTSGDDGKIE

DFEPRPSATSYDGDLKS

EFEPRPSVTKYDGDYYKNKKSQLNDEFEPRPSVTRYDDGSYKNGKLYVSAEFEPRPSATKYDGDGYKMAKLPVNSEFEPRPSVTKYDGGDYKNKKSRVNSEFEPRPSVTRYDDGSYKSGKLYVSVEFEPRPSVTKYGGDDYKMVKLPVNSEFEPRPSATKYDGHDYKNKKLHVNEEFEPRPSATKYNE

## *C. arietinum* ST2 (CarST2). Full length, 11 repeats, 26 amino acids. Subtype IIa

MRPALAFFPLLLFSFVAMVES

RKDPAEYWKMVMKDKDMPEAIQGLLNTNSKKGLKHCDEKLVKDTQVIVEEKVYIE

DLEPRPNISAYEGNDVDNKEKKKDIKDFEPKSNISAYEDNVIDYNEKKEDIKDFEPRPNISAYGDNEIDSSEKKESVKDFEPRPNISAYDNNDIDVKEKKEALEDFEPRPNLSAYGDNEIDSSEKKESVKDFEPRPNISAYDNNDIDVKEKKEALEDFEPRPNLSAYGDNEIDFKEKKETLEDFEPRPNISAYGDNEIDFKEKKEAFEDFEPRPNISTYGDNGIGSKKKKEVVKDFVSRPNISTYENNNIDDEINEKFKVDFEPRPSATKYDA

## *Cichorium endivia* ST1 (CenST1). C-terminal, 4 repeats, 24 amino acids. Type I

GGDKAASTLYLELDSHLSFLVLFSLVMISITAYARPNPEEYGQDWRVRGGSLVSPHPTKKSPCNTLTEASNQRMSSNE

DFEPRPNISVYDNGASLKSKKTFDE

EFEPRPSTTAYGGASLKDKKTFDEEFEPRPSTTAYGSASLKDKKTFEEEFEPRPSTTSYGV

## *Citrullus lanatus* ST2 (CilST2). Internal, 6 repeats, 23 amino acids. Subtype IIa

LVFINVLLLVLFAITIESRHEPGEHHWRNLINHKLLFHEANEDLIRSDPNSLLSKKKMDDCTENLKVEDGKL

FVEPRPQATFYRGVVKAKLFAK

DIKPRLDNTKTKLFVE

DVEPRPNVSFYPNEDVKTKLFVEDIEPRPNVSFYPDDDTKTKLFVKDVEPRPNVSFYPDDDTKTKLFVEDIEPRPNISFYPDDDTKTKLFVEDVEPRPNVSFYPDNLKAKEHSADDAHHHGEADIQMVQA

## *Citrus jambhiri* ST2 (CjaST2). Internal, 6 repeats, 26 amino acids. Type III

MLRKDLRDYWRIVAKNQDMPGESTQGLIPEDQASTVSKTTAYCHTHEDSEHTMEKRFVNN

NFELMPDVSIYDNGIKLTKQKAFVK

DLELMPDVSIYDNGIKPKDKQRSFAKNFELMPDVSIYDNGIKPTKQKAFAK

HFEFLPDVSIYDNGIKPTKQKFFAKDFELMPDVSIYDNGVKLAKQKFSP

RTSNSCLMFHYNNGVELAKQKAFPKA

## *C. paradisi* x *C. trifoliata* ST2 (CptST2). N-terminal, 8 repeats, 25 amino acids. Type III

MNIKAFFVLSTLLSLLILFANMIDA

RKDLRDYWRIVVKNQDMPGESTQGLIPEDQASTVSKTTAYCHTHEDSEHTMEKPFVNK

NFELMPDVSIYDNGVKLTKQKAFVK

DLELMPDVSIYDNGIKPKDKQRSFAK

NFELMPDVSIYDNGIKPTKQKAFAKHFEFLPDVSIYDNGIKPTKQKFFAKDFELMPDVSIYDNGVKLAKQRFSAKDFELMPDISIYNNGVELAKQKAFAKGFELMPDVSIYDNDIKPSKKSSYAKDFELRADISIHEPTEQK

## *C. reticulata* ST2 (CreST2). Full length, 6 repeats, 25 amino acids. Type III

MNIKAFFVLSTVLSLLLLFANMIDA

RKDLGDDWSIVVKNQDIPGESTQGLIPEDQGSTISKTKAYCHTHDDSEHTMEKPFVNK

KFELMHDVSIYDNGIKPKDQQRSFAK

NFELMPDLSIYDNGIKPTKQKAFAKNFELMPDLSIYDNGIKPTKQKFFAKNFELMPDISIYDNDIKPTKRSSYAKDFELKADISIHEPTEQKSVVS

STDLQPDDTIYHN

## *C. sinensis* ST2 (CsiST2). N-terminal, 7 repeats, 25 amino acids. Type III

MNIKALFVLSTVLSLLLLFANMIDA

RKDLRDYWRIVVKNQDMPGESTQGLIPEDQASTVSKTTAYCHTHEDSEHTMEKPFFNK

NFELMPDVSIYDNGVKLTKQKAFVK

DLELMPDVSIYDNGIKPKDKQRSFAKNFELMPDVSIYDNGIKPTKQKAFAK

HFEFLPDVSIYDNGIKPTKQKFFAKDFELMPDVSIYDNGVKLAKQRFSAKDFELMPDVSIYNNGVELAKQKAFAKGFELMPDVSIYDNDIKPSKKSSYAKDFELKADI

## *Codonopsis lanceolata* ST2 (ClaST2). N-terminal, 6 repeats, 25 amino acids. Subtype IIa

MESPFTLITLLSLLLIASTIDA

RPDPRFLGDVVEDTPRPGAFEGIFHKGTSVLHVPNKNSDCHTSNHGIGRATRNGHKPLVK

DFEPRPNVSAYDDEKLTEKKPEEDQDFKPRPNVSAYDNDVRPEEKKAPVRDFEPRPNVSAYDNDVHPEEKKEPVRDFEPRPNVSAYDNDVHPEEKKAPVRDXEPRPNVSAYDNDVHPEEKKAPVRDFEPRPNXSAYDNDVHPEEKK

## *Coffea arabica* ST2 (CoaST2). Full length, 2 repeats, 27 amino acids. Type not determined

MASPVALIALISLVLFAGITEA

RKDPGEYWQGVAARNDQALLEAIPHLVRIDSTLSDTKKITTASDCHTSNKKDSVAEAANNKKSVFAT

DFEPRPNLSAYGDDAKLKKKEEKTFTKDFEPRPGASFYAN

## *Corchorus capsularis* ST2 (CocST2). N-terminal, 4 repeats, 26 amino acids. Subtype IIa

MKSFPSFLGFLSLLMLADTIAAA

RKDGGEYWRVAVMKDESIPEAIESALVPVNAAAASSSGDKTNCHDLPSNIEIKEEKIFVE

DFELPRPNNYNSVEGGVTKERSFAK

DFEPRPNLSAYGDDGDLKEEKKSFAKDFEPRPNLSAYGGDGDLEEEEKSFAKDFEPRPNLSAYGDDGYLKRGGKSLLVIG

## *C. capsularis* ST3 (CocST3). Internal, 3 repeats, 26 amino acids. Subtype IIa

TYGEEGIDVKEQFICVK

DFEPRPNLSAYGDDVVDLKEKKKFAKDFEPRPNLSAYGGDDSDLEEEKQIIKDFEPRPNLSAYGDDGI

## *Cucumis melo* ST2 (CmeST2). Internal, 8 repeats, 23 amino acids. Subtype IIa

RPNVSFYPDDSTKTRLFVE

DVEPRPNVSFYPDDETKTELFAKDVEPRPNVSFYPDGETKTELFAKDVEPRPNVSFYLDDDTKTKLFAKDVEPRPNISFYPDDDTKTKFFVEDVEPRPNVSFYPDDETNTKLFAKGVEPRPNISFYPDDDTKTKRLVQEIELQPNVSFYPDDDTKTKLLVEDIEPRPNVSFYPNNLKAKEQLSADSHRGEAGLQVAQA

## *Cucumis sativus* ST1 (CsaST1). Internal, 9 repeats, 23 amino acids. Type I

EQGQGFTK

DIEPRPSVTFYPNDESKDKLFTKDIEPRPSLTFYPNDESKDRFFTKDIEPRPSATFYPNDDTKNKLFTKDIEPRPSATFYPNDESKGRFFTKDIEPRPSATFYPNDDTKNKLFTKDIEPRPSLTFYPNDESKDRFFTKDIEPRPSATFYPNDDTKNKLFTKDIEPRPSATFYPNDDANKKFFTKDIEPRPSVTFYPNNDSKNKLFIKNIESRLSTTE

## *Cucumis sativus* ST2 (CsaST2). Internal, 10 repeats, 23 amino acids. Subtype IIa

MKDKMDDCTETLKVEDGKLFIEPRPQATFHGDVQTKILSKDLEQRPSVSFRPDDTRTKLFVE

HIELSPSIKFYPHEIKAKLAK

DIDVPPRTLIYLNDIKSNFFIKDIERQLRARFYRDDNKRKLAK

DIEPRPNVSFYPDDTKTKLFAE

DVEPRPNVSFYPDDETKTKLFAEDVEPRPNVSFYPDDDTKTKLFVEDVEPRPNVSFYPDDETKTKLFAEDVEPRPNSFFYPDDDIKTKLLVQEIEPRPNVSFYPDDDTKTKLFAEDIEPRPNVSFYPDNLKAKEQLSAHSHHGEAGLQVAQA

## *Cyamopsis tetragonoloba* ST1 (CteST1). N-terminal, 6 repeats, 29 amino acids. ST-like

MRLSLSLIPLLFVSFLAMESES

RKDEGEYWKIVMKDEKMPEAIQGLLQSSSRIKPGLNNLNTQVSKEEKVVAK

EFKVGSNALLLYSAYAENGNAKEEQKYVK

EFEPGSSVFLFYEPYAKNGDAKEDNTYVK

EFEPGSNALLLYTTYAKNGDAKEDNTYVK

EFEPGSNALLLYTTYAKDGDSNEKKYVKEFESESNALLLYTAYAKDDNSKEKKYMKELEPRSNAFIGYTAYAKDGNSKEKKY

## *Eucaliptus grandis* ST2 (EgrST2). Full length, 3 repeats, 24 amino acids. Subtype IIa

MSYQVSFTIIVAVLIFTATTDA

RKDPGEYWVSIMKEEPMPEAIEGLLHVSNSASDQPRLTSLPKKKDADYHGDNKLEDDKQFAG

DFEPRPNISAYGDDAKLQEKKFAEDFEPRPNISAYGDDTRLKEKKFVEDFEPRPNISAYGQD

## *E. grandis* ST3 (EgrST3). N-terminal, 3 repeats, 24 amino acids. Subtype IIa

MSYQVSFTIIVAVLIFTATTDA

RKDPGEYWVSIMKEEPMPEAIEGLLHKKDADCHGDNKLEDDKQFAG

DFEPRPNISAYGDDAKLQEKKFAEDFEPRPNISAYGDDTRLKEKKFVEDFEPRPNISAY

## *E. globulus* ST2 (EglST2). C-terminal, 3 repeats, 24 amino acids. Subtype IIa

WVSIMKEEPMPEAIEGLLHVSNSASDQPHLTSLPKKKDADCHGDNKLEDDKQFAG

DFEPRPNISAYGDDAKLQEKKFAEDFEPRPNISAYGDDTRLKEKKFVEDFEPRPNISAYGQD

## *Fragaria x ananassa* ST2 (FanST2). N-terminal, 2 repeats, 25 amino acids. Type not determined

MNSLSAIFALLSLLLFAIIVES

RVGPGGYEKIVIEKQPMPEDLWKLVKEKFELNSADNLSDDKAEKADCHENGKPKIEFEVA

EFEPRPNLSVYPDDSEVTGSRTLEDEPESKAIGNMDSKDNGPKDQLPFEAKAKNHAFEE

DKEPRPDVTVYND

## *Glycine max* ST1 (GmaST1). Full length, 7 repeats, 24 amino acids. Type I

MRPALALLPLLFAFLKLAATTES

RKDPGEYWKMIMKDQEMPEGLQGLVSFQSENNPKTQEQLGKGSKNHCEESLVTNTQVNS

DFEPIPSVTKYDDLEFKSSIIKNDDFESRPSVTKYDDFELKSSVTKNNDFEPRPSATKYDDFELRSSVTENDDFEPRPNISKYDDFELRSSVTENDDFESRPSVTKYDDLELKSSITKNDDFEPRPNISKYDDFASRSNTKY

DSEPKASATKYSD

## *G. max* ST2 (GmaST2). Full length, 13 repeats, 26 amino acids. Subtype IIa

MRRALALLPLLFLFVAIGES

RKDGGKYWKMNTRDHQMPKKFKALLNQNFEIQSEKILKTKEQILEGSKHDCEKSLVKNTHTTILE

DFEPISSVPIYRNDDIYAKEENKVVKNFEPRPNVSAYGDNDVDAKPKKNVIKDFEPRPNVSAYGDNDVDAKPKKNVMKDFEPRPNVSAYGDNDVDVKPKKNVMKDFEPRPNVSAYGGNDVDAKPKKNVMKDFEPRPNVSAYGGNDVDAKPKKSVTKDFDPRPNVSSYNDNDIEAVEEKKIMKDFEPIPKVSTYGDDDVNTKEKKKVMKDFKPRPNVSAYGDNNVSAKEKKKVMKDFEPRPNVSAYGDNNVGAEKKKKIMKDFEPRPNVSAYGGNNVDEEAKKKVTK

DFESRPNISAYGNNGVDAKKKKKVVDNDNIDAQGKPPE

DFEPRPNISTYDE

## *Glycirrhiza uralensis* ST2 (GurST2). N-terminal, 4 repeats, 26 amino acids. Subtype IIa

MRPAIALFPLLFLLFVTTAAS

RKDVGEYWKRVMKDQDIPEEIQGLLNVNSEIQYEKNLKTQKLPFEGNTHNCEESPLVKNTQVTIDEKNKKVMQ

DFEPRPNVSAYGDDEIEAKEKKKAVGDFEPRPNVSAYGDDEIEAKEKKRVVK

DFEPRPNISAYGDDEIEGFEAKEKKRVVKDFEPRP

## *Gossypium hirsutum* ST2 (GhiST2). Full length, 3 repeats, 27 amino acids. Subtype IIa

MKSFLSFFAFLSLLLFVDTIAAA

RKDAGEYWGAVTKDQPMPEALQELVRIEAAVVSPDEKTKCHTSGNIELKEEKIIVNE

EFEPRPNVSAYGDDANLKGDKSSSFAEDFEPRPNVSAYGGDDAGLKGQKESFTKDFDPEPNVSAYNN

## *Helianthus argophyllus* ST1 (HarST1). N-terminal, 7 repeats, 23 amino acids. Type I

MKSLHSFLVFFSLTMIVFIIKA

RPDSGEYWQNLIVQKSDPVSPLEGNKAHCHTLKETTLKASGFEDESPYGNIKDKDYEDITHLNEKKAFNT

DFEPRPSATSYVTGVSDKKSYDTKFEPRPSATSYVTGQNGKKTFNTDFEPRPSATSYVTGLNGKKTFDTGFEPRPSATSYVTGLNGKKTFNIEFEPRPSATNYVTGQNGEKTFDTGFEPRPSATSYVTGLNGKKQFDTEFEPRPSATSYVASLNGKKTFNIDFKPRPS

## *H. argophylus* ST2 (HarST2). Full length, 9 repeats, 25 amino acids. Subtype IIa

MESSLAFLVLLSLIMTAIMNIDA

RPHPEEYWQDSFVRRGTSVSPQPIKKSHCHTLAKASNHISSGHD

DFEPRPNISVYDEDTSLKGFKE

EFEPRPNISVYNNNIGLKGKNKVDEELEQTPDISIYDNDTSLRGKKNVDEDFETSPNISVYDNDAGLKGKKNVDEEFEPRPNISVYDNDTGLKGKKNVDEVFEPRPNISVYDNDAGLKGKKNVDEEFEPRPNLSVYDNDTGLKSKKNVDEEFEPRPNISVYDNDLRMKGKMTSKEDFEPRPNISVYEG

## *H. ciliaris* ST1 (HciST1). Full length, 10 repeats, 23 amino acids. Type I

MESLRSFLVFFSLTMVVFIIEA

RPDSGECWQNLNEKKAFNT

DFEPRPSATSYVTGVSDKKSYDTKFEPRPSATSYVTGLNGKKTFDTGFEPRPSATSYVTGLNGKKTFNIDFEPRPSATSYVTGQNGKKTFDTRFEPRPSATSYVTGLNGKKPFDTGFEPRPSATSYVTGQNGKKTFDTGFEPRPSATSYVAGLNGKKTFNIDFKPRPSATSYVAGQNGMKTFDT

NFEPRPSATVYDNRVNTKDNAPFDK

DLEPRPSATSYDI

## *H. ciliaris* ST2 (HciST2). N-terminal, 9 repeats, 25 amino acids. Subtype IIa

MESSLAFLVLLSLIVTAIMNIDA

RPHPEEYWQDSFVRRGTSVSPQPINKSHCNTFAKAINHISSGHD

DFEPRPNVSSYGNDANPDGNKK

DFEPRPNVSVYVEDTSLKGKENFKEEFEPRPNVSVYDNNIGLKGKSKVDE

EFEPRPNVSVYDNTSLKGKKNANE

EFEPRPNVSIYDNDTGLRSKKNVNEEFEPRPNVSVYDNDAGLKGKKNVDEAFEPRPNISVYDNDAGLKGKKNVDEEFEPRPNVSVYDNETGLKSKKNVDEEFEPRQNVSIYDNDLRL

## *H. ciliaris* ST3 (HciST3). Full length, 8 repeats, 25 amino acids. Subtype IIa

MESSLAFFLLLSLIMGATNIDA

RPHPQDWQDSFIQKGSSVSSQPIEKSHCNTLSKARNHISPAKN

ELESRPNTSSYVNDHMNPNDDNN

DFEPRPNISVYDNDTGLKGKKNVDEEFEPRPNISVYDTDTNLKGQKNVDEEFEPRPNISVYDNDTDVDE

EFEPRPNISVYDTDTNLKGQKYVDEEFEPRPNISAYDNDTGLKGKMTLNKEFEPRPNISVNDE

EFEPRPNISVYEG

## *H. paradoxus* ST1 (HpaST1). N-terminal, 7 repeats, 23 amino acids. Type I

MESLHSFLVFFSLTMVVFIIEA

RPDSGEYWQNLIVQKSDLVSPLEGNKAHCHTLKETTLKASSFDDESPYGNIKDKDYEDITHLNEKIAFNT

DFEPRPSASSYITSVSDKKSYDPKFESRPSATSYVTGLNGKKTFNIDFEPRPSATSYVTGLNGKQTFDTGFQPRLSATSYVTSLNGKKPLGTELEPRPSATSYVVALNGKKTFNIDFKPRPSASSYVAGQNGMKTFDTNFEPRPSASVYDNR

## *H. paradoxus* ST2 (HpaST2). N-terminal, 7 repeats, 25 amino acids. Subtype IIa

MESSLAFLVLLSLILTAIMNIDA

RPHPEEYWQDSFVRRGTSVSPQPIKKSHCNTFAKASNPISSGHD

DFEPRPNVSSYGNNDVNPYGNKK

DFEPRPNVSVYDEDTSLKGKENFKEEFEPRPNVSVYDNIIGLKGKTKVDEEFEPRPNVSVYDNGTSLKGKKNANEEFEPRPNVSIYDNDTGLRSKHNVKEEFEPRPNVSVYDNDTGLKGKKNVDEAFEPRPNVSVYDNDSSLKRKTNVD

## *H. petiolaris* ST2 (HpeST2). N-terminal, 9 repeats, 25 amino acids. Subtype IIa

MESSLAFLDLLSLIMTAIMNIDA

RPHREEYWQDSFVRRDSSVSPQPIMKSHCNTFAKASNHISSDHD

EFEPRPNVSSYGNDVNPDGNKK

DFEPRPNVSVYDEDTSLKGKENFKEEFEPRPNVSVYDNNIGLKGKSKVDAEFEPRPNVSIYDNDTSLKGKKNANEEFEPRPNVSIYDNDTGLRSKNNVNEEFEPRPNVSVYDTDTGLKGKRNVDEAFEPRPNVSVYDNDAGLKGKKNVDEEFEPRPNVSVYDNETGLKSKKNVDEELETRPNVSIYDND

## *H. tuberosus* ST1 (HtuST1). Full length, 10 repeats, 23 amino acids. Type I

MESLHSFLVFFSLTMIVFIIEA

RPDSREYWQNLIVQKSDPVSPPEGNKAHCHTLKETTLKASSFEDESPYGNIKDKDYEDITHLNEKKALNT

DFEPRPSATSYVTGVSDKKSYDTKFEPRPSATSYVTGLNGKKSFNIDFEPRPSATSYVTGLNGKKTFDTGFEPRPSATSYVTGLNGKKTFNIDFEPRPSATSYVTGQNGKKTFDTGFEPRPSATSYVTGLNGKKPFDTEFEPRPSATNYVASLNGKKTFNIDFKPRPSATSYVAGQNGMKTFDTNFEPETSATVYHNRVNTK

DLNPRPSANSYDI

## *H. tuberosus* ST2 (HtuST2). Full length, 6 repeats, 25 amino acids. Subtype IIa

MESSLAFLLLLSLIMGATNIDA

RPHPQDYWQDSFVQKGSSVSSQPIEKSHCNTLSKARTHISPVKN

ELESGPNTSSYVNDHMNPNDDNN

DFEPRPNISVYDNGTDLKGKKNVDEEFEPRPNISVYDTDTNLKGQKNVDEEFEPRPNISVYDNDISLKGKMTLNKEFEPRPNISVNDK

EFEPRPNISVYEG

## *Jatropha curcas* ST2 (JcuST2). Full length, 11 repeats, 22 amino acids. Type III

MKFFFAFLSLFSLILFSITINA

RKDVGEYWRGEMKDQPLPEALQRLRASPVSSTSTEEKVDVFVYHNDDAKFKE

NFELRPDATIYHNDARLKARKP

YFVTRDDVTIYHDDMRFKE

KFEPRSDVIMYHNDAKLKSQEP

FPETRNDVTIYHDDVEIKE

KFELRPDATIYHNDATLKPQKP

YFETKDDATIYHDDVAIKE

KFELRPDATIYHNDATLKPQKP

YFEIKDDATIYHDDVGSKK

EFELRPDVFVYHIDARPKARKS

YFETKDDVTIYHDDIGLKKKLFSN

DFESKPDVTVYSE

## Juglans hindsii x J. regia ST2 (JhrST2). Full length, 3 repeats, 27 amino acids. Subtype IIa

MMNSRIAWLVMALIFSVLLIATDHSIEA

RKDPGEYWISVMKEEPMPEALQVLVPLDSSPSQLNKNADDCHTPELGAEINKLDQLVEDFKHKADHDHHKQRFVK

DFEPRPNVSAYDGDNDVGHESEKKFIKDFEPRPNVSAYTDDHVHSSKERAFAEEFDQQTPDATIYHE

## *J. hindsii x J. regia* ST3 (JhrST3). Full length, 3 repeats, 27 amino acids. Subtype IIa

MMNSRIAWLVMALIFSVLLIAADHSIEA

RKDLGEYWINVMKEQPMPEALQVLVPLDSSPSQLNKNADDCHTSELGVEINKLDQLIVEDFKHKADHDHKQRTFVK

DFEPRPNVSAYDGDDDVGHESEKKFIKDFEPRPNVSAYTDDHVHGSKERAFAEEFDQQTPDVSIYHE

## *Lactuca sativa* ST2 (LsaST2). Full length, 4 repeats, 25 amino acids. Subtype IIa

MESRLSFFALFFLIMITVTVYA

RPNPEEYQQDSHVRQGSLVSTDPTKRSPCNQRKSSNE

DFQPRPNISVYDNSAGLKDKKIFPDDFEPRPNISMYENGASVKGKKMSDEEFEPRPNISVYDNSATLKGKRTFDEEFEPRPSATAYKG

## *L. serriola* ST2 (LseST2). Full length, 4 repeats, 25 amino acids. Subtype IIa

MESRLSFFALFFLIMITVTVYA

RPNPEEYQQDSHVRQGFLVSTNPTKRSPCNQRKSTNE

DFEPRPIMSVYDNSAGLKDKKILPDDFEPRPNVSIYENGASVKGKKMFDEEFEPRPNVSVYDNSGSLKGKRTFDEEFEPRPSATAYKG

## *L. virosa* ST2 (LviST2). Full length, 4 repeats, 25 amino acids. Subtype IIa

MESHLSFLALFFLITITVTVYA

RPNPEEYQQDSHVRGGSLVSSDPTKRSHCHQRKSSNE

DFEPRPNISVYDNSDGLKDKKMFPDDFEPRPNLSIYENGASVKGKKMFDEEFEPRPNVSVKDNNASIKGKKTFDEEFEPRPSITAYKG

## *Lens culinaris* ST1 (LcuST1). Full length, 5 repeats, 26 amino acids. Type I

MRPAHALLPFLILFLFSVTTES

RKDLKEYWQTVMKDEEMPEGIQGLLQLKSEMEPLKNSMVGKGNCDKHPITKTQNVKEKQVVSE

EFEPRPSATKYDGDESYENKKLPVNGEFEPIPSVTKYDGDESYKSMKLPMNGEFEPIPSVTRYDGDDGYKSMKLPVNDEFEPRPSATKYDGNDYKTMKLHVND

EFEPRPSATKYNE

## *Lotus japonicus* ST1 (LjaST1). Full length, 7 repeats, 25-26 amino acids. Type I

MRPALPLLPFLFLFLIAATVES

RKDQGEYWKMIMKDEEMPEGIQGLLQLKSEIKPGKNSEHKCDEEHVVTNNEYIIEKKVSTE

ELEPRPNISAYDDDHVDTKANKKYMEDLEPRPNISAYDDDHVDTKGNRKYMEDLEPRPSVSAYSEEIVK

TFEPRPSVTAYIEHDHKGEKLALNSEFEPRPSVTSYREQIVK

TFEPRPSVTAYIKDGHKGEKLMVANEFEPRPSATKYND

## *L. japonicus* ST2 (LjaST2). N-terminal, 3 repeats, 26 amino acids. Subtype IIa

MRSALALLPLLFLFLVASTVES

RKDQGEYWKMIMKDEEMPEGIQGLLQLKSEIKPGKNSEHKCDEEHVVTNNEYIIEKKVFTE

ELEPRSNISAYDDDHVDTKGNKKYMEDLEPRPNLSAYDDDHVDKKGNKKYMEDLEPRPNLSAYDDDHIDTKGDKKYMERIWNLGQTFQLITTK

## *Lupinus albus* ST1 (LalST1). N-terminal, 8 repeats, 16 amino acids. Type I

MRPSLTLLPLLFLFLIVANVES

RKDLVHIMKDQKLEDKTQYLLKLKVDIQSENNLKIMEKHVEDFMQKCEEPPITNTQVTIEKKVLTE

DFEPRPNSVYTNENVDMKENKEFDKDFEPRPSATRYNPVNN

DFEPRPSLTRYNPVNTKFEPRPSATRYNPVNSDFEPRPSATRYNPVNTEFEPRPSATRYNPVNTEFEPRPSATRYNPLNSDFEPXPSATRYK

## *L. albus* ST2 (LalST2). Internal, 7 repeats, 25 amino acids. Subtype IIa

GSAYGDNDVDAKENEVVK

DFEPRPNISAYGDNDVDAKEKEVVKDFEPRPNISAYGDNDVDAKEKEVVKDFEPRPNISAYGDNDIDAKKKEGVKDFEPRPNVSAYANNDINAKKKEVVKDFKPRPNVSAYGDNNVNAKKKEGVKDFEPKPNISEDDCEEKKKIMT

KFELRSN

## *Manihot esculenta* ST2 (MesST2). N-terminal, 5 repeats, 30 amino acids. Type III

MKSLFALVVPLFLLLSILSSINA

RKDLDEYWKGVTKDQPVPEAMQKLLQASDEKTNCHTTKTLEPNADLKIYHNDDFLKEGKSSFLQ

QFEPRPDVSIYHNDIEKLLNKNPFVK

EFEPRPNVSIYHNNIGLKTKKPLDDSAFNKEFESKPDVSIYHNNIDLKLKNSPDESSYNKEFEPTLAVTIYHNDIDLQAKEQSDERLVGKEFEAKPDVTIYHNDKNFTEA

## *M. esculenta* ST3 (MesST3). Full length, 7 repeats, 30 amino acids. Type III

MKSFFALLSLFLLLLILSSTYA

RKDVGEYWRGDIKAQSLPEPIQELLHASPTSSASNRKTSCGMSK

NFEPRPDVSIYHDHIGLQKKNPMHETYSLQKFKSRPDVTIYHNDIVLEAEKQWNEKSFLK

KLEPRYDVSIYHNDDDNDVPDGFKGKKTLHEKSF

VNKFEPDVSIYHNDIILEVQK

ELEPRPDVTIYHNDLPNDFKGKKPLSEKSFVT

KFEPRPDVSIYHNDAGFKAEKPSEEKSFLSNFEGKEDVTIYHEKL

## *M. esculenta* ST4 (MesST4). Full length, 3 repeats, varied amino acids. Type III

MKSFFASLPFYLLLILSTTYA

RKDEGEYWRGVMKDQPLPEPIQELLQASPTSSDGKTDSGMSK

NFEPIPNASIYHDDIGLQTKNPLSQTHSLQNFEPIPDVSIYHDDIGKKPLNKESFAK

KFEPRPDLTIYHNDE

## *M. esculenta* ST5 (MesST5). N-terminal, 3 repeats, varied amino acids. Type III

MKSFFASLPFYLLLILSTTYA

RKDEGEYWRGVMKDQPLPEPIQELLQASPTSSDGKTDSGMSK

NFEPIPNASIYHDDIGLQTKNPLSQTHSLQNFEPIPDVSIYHDDIGKKPLNKESFAK

KFEPRPDLTIYHNDEVFKEEKPSEEKSFTKDD

## *Medicago truncatula* ST1 (MtrST1). Full length, 12 repeats, 26 amino acids. Type I

MRPALAVLTLLFLFLFAATIES

RNDLKEYWKTIMKDEEMPEGIQGLLQLKSEIEPMKNSKAQEQLAKGKCDEHPATNTLNIIKKKVVTE

EFQPRPTISAYGDDKIDIKVTEKHIK

DFEPRPSGTRYDGDGYKSEKLRVYS

EFEPRPSATKYDGDGEGYKTIKLPGND

EFEPIPSVTRYDGDGYKSMKLSGNDEFEPRPSVTRYDGDGYQSTKLSVNNEFEPIPSVTKYGGDGYKNTKLSVNSEFEPIPSVTSYGSDGYKSMKLPVNDEFEPRPSATKYDGDGYQSTKLSVNSEFEPIPSVTSYGSDGYKSMKLPVNDEFEPRPSATRYDSDDYRKMKLPMNDEFEPRPSVKKYDGDNNKSKKLHVNDEFEPRPSATKYND

## *M. truncatula* ST2 (MtrST2). Full length, 11 repeats, 26 amino acids. Subtype IIa

MRPALALFPLLLFLFVAIVET

RKDLGQYRKLVMKDEDMRKEIQGLLYTETKKNLKTPKQCFKGFKHDCEEPFVIDTQVTIENDIIRAKRKKGVTK

NFEPRPNVSKYEDNDINAEEKKRVVKDFEPRPNVSAYGANEIDAKDKKGVIDDFEPRPNVSAYGDNDVEAKKKKGITEDFEPRYNVSAYGDNGVEVKGKKKVVKDFKPRPNVSAYGDNGIDVKENKGANT

DFEPRPNYVSAYGDNNIDVTENKGANK

DFEPRPNVSAYGDSDIDVKENKGVSKDFEPRPNVSTYGNNDIDVTENKEVNKAFEPRANVSAYKNNDIDVMKKKNVTKDFEPRSNVSVYGDNNI

DVEPRPSATKYDA

## *M. truncatula* ST3 (MtrST3). Full length, 15 repeats, 26 amino acids. Subtype IIa

MRSALALLPLMLILFVEIVES

RNNLGEYWKLFMKDQNMPEEIQGLLSANTKSNLKTLEKKVFGE

DIEPRRNISAYKNDGSKQDVK

DFEPRSNISAYGDNDIDVKEKNGAIKDFEPRPNISAYGENVIDADEKKKPVKDFEPRPNISAYGDNDIDVKENKGATNDFEPRPNISAYGDNVIDDNKMKKPVEYFQPRPNISAYGENDIDVKKSK

DFEPRPNISAFGDNDIDVNEKKGDTKDFEPRPNISAYGDNAIDDDEMKKPIKDFKPKPNISAYGDNVIDADEMKKPVE

GFEPRPNISTYGDNVIDVDEMKKQQVK

DFEPRTNISAYGDNDIDVKEKKGVTNDFEPRPNISAYGDNVIDANKMKKPVDDFQPRPTISAYGDNAIDNSKTNEVIKDFESRPNISAYGNNEVDTKKKEKAAKDFEQRPIISA

## *M. truncatula* ST4 (MtrST4). Full length, 17 repeats, 26 amino acids. Subtype IIa

MRTALALLPLMLFLFVAIVES

RNDLREYWKLVMKNQDMPEEIQGLLNANIKSNLNTQEKAFKE

DLESRPNISAYENDGTNTKENSKDVKDFEPRPNISAYGDNDIENKEKKGTVKDFEPRPNISAYGDNAIDVKEKKKVVKDFEPRPNISAYEDNVVDVKKKKGVTKDFEPRPNISAYGENDSDVKQKKQTINDFEPRPNISAYGENDIDVNEKKAAAKDFEPRPNISAYGDDVIDVKEKKKAVKDFEPRPSISACGDNVIDVKKKKGVTEDFEPRPNISTYGENDIDVKEKKQATNGFKPRPNISAYGKNDIDVHEKKAAGKDFEPRPNISAYGDNAIDAKEKRGATNDFEPRPNISAYGDNDIDVNKKEGAIKDFEPRPNISAYGENDINDNETKKPVEDFEPRPNISAYGDNDIDDRKKNNVISDFEPRPNISAYGNNDIDTKEKEKAAKDFEPRPNISAYGNNDIDTKEKEKAAKDFEPRPNISAYGEK

## *M. truncatula* ST5 (MtrST5). Full length, 14 repeats, 26 amino acids. Subtype IIa. Theoretical intron splicing (putative mistake)

MRAALALLPLLLFMMKDQDMP

EEIQGLLNANTKSNIMTMDHYSKGSKLGCEELLVKDTLVTNENVFEE

NIEPRPNISSYENDETNIKENKKDTKDFESRPNISAYGDNDINATEKNGDTKDFEPRPNISAYGDNEIDVKEKKGAAKDFEPRPNISAYGDNDIDVKEKKGATKDFEPRPNISAYGDNDIDVNEKKGAAKDFEPRPNISAYGDNDIDVNEKKGATKDFEPRPNISAYGDNEIDVKEKKGATKDFEPRPNISAYGDNDIDVNEKKGAAKDFEPRPNISAYGDNDIDVNEKKGATKDFEPRPNISAYGDNEIDVKEKKGATKDFEPRPNISAYGDNEIDVKENNGATKIFEPRPNISAYDNNDIDTKEKEKAAKDFEPRPNISGYGNNDIDADKNEEFTNDFEPKPSVTKSDH

## *M. truncatula* ST6 (MtrST6). Full length, 17 repeats, 26 amino acids. Subtype IIb

MRPALALLPLLLFLFATTMES

RKDPGTHSKLVMKDRDTKEDIQVLLNNNAKKN

LKTQKPNDLLYGDNEIDAKANKGANKDFEPRPNFFLYGDNAIDAKENKVANKDFKPRPNFFLYGENGVDAKENKRSGKDFEPRPNFFLYGENGVDAKENKRSGKDFEPRPNFFLYGENGVDGEENKEATKDFESRPNFFLYGKKEVNTKENRGVNKDFEPRPSFFLYGEKRVDTEKNKGATNDFEPRPNFFLYGENGVNAKENKGATKDFNPRPNFFLYGKNGVDAKENKGFIKDFEPRPNFFLYAENGVDAEENKEATKDFEPRPNFFLYGGKRVDTKENKGSIKDFESRPNFFLYGEKGVDTKENKGTAKDFEPRPNFFLYGENEVDAKKNERTTKDFEQKSNFLLNGDNEIDAKENTVGTKDIEPRPNFFLYRANGNDAKEDKVITDDFKPRPNFFLYGDHVTDVKEKK

DFEPKSSVAKYDA

## *Mimulus guttatus* ST2 (MguST2). Full length, 4 repeats, 25 amino acids. Subtype IIa

MVSIYALSGLLCISLFASFTNA

RKDPGAYLLQDFGKVTSTISNEEIDCHALGKASTPKPKTSYTK

EFEPRPNISAYTDDEKTDENKSFVKDFEPRPNISAYTDDDKTEENKSFVKDFEPRPNISAYTDDEKTEENKSFVKDFEPRPNISAYTD

## *Nicotiana benthamiana* ST1 (NbeST1). Full length, 6 repeats, 26 amino acids. Type I

MKLPVALILLFSLALYASSTDA

RKDPGEYWRAVMKDEPMPEAIKHLMPRHSVPLSKEKTDCYTSSSVGGE

ASEPKPNLYVYHNDANLKEAEKSLFTRDFEPRPSATGYHDDDVGLKEKSLFTR

DFEPRPSLTGYHDDDVGLKEKSLFTRDFEPRPSVTGYHDDDVGLKEKSLFTRDFEPRPSVTGYHDDDVGLKQEKSFAEDFEPRPNLSVYHD

## *N. sylvestris* ST1 (NsyST1). C-terminal, 3 repeats, 25 amino acids. Type I

GTTSYHDDTGLKQEKSFAE

EFEPRPSLTSYHDDAGLKQEKSFAEEFEPRPSITTYLDDAGLKQEKSFAEEFEPRPNVSVYHD

## *N. tabacum* ST2 (NtaST2). Full length, 3 repeats, 25 & 26 amino acids. Subtype IIa

MKLHVALILLFSFALYTSSTNA

RKDPGEYWRAVMNDEPMPEAIKHLMPQHSVPLSIEKTDCYTLPSTGGE

AFEPRPNLSVYHDDAKLKEAEKLLFMKDFEPRPTITGYHDDDAGLKQENPFTE

DFKPRPNASVYHD

## *Oxytropis campestris + O. splendens* ST2 (OcsST2). Full length, 3 repeats, 25 amino acids. Subtype IIa

MRSTALALLAFLFLVLLATTIES

RKDLRDNWKTKDEEISGRIEGLPQLRYEIEPKRNSVSTQEQLAKGLEHNCEEKVFAK

EFEPRPNISAYDQDDVDTKENKKFTKDFEPRPNISAYDHDHVDTKENKKFVKDFDPRPNISAYSDEDIK

## *O. campestris + O. splendens* ST3 (OcsST3). Full length, 4 repeats, 26 amino acids. Subtype IIa

MRSTLALLSLLIFLFAAIVES

RKDPGEYWNMVMKDQNIPEEIQGLHNTNSEIKFKENLKTLKHSHESSEHNCEEPLVKDTQITIEKVFVE

EFEPRPNISAYDGDEIDVDEKKKVVKDFEPRPNISAYGDNDVDAKENKKVTSDFEPRPNISAYGDNDIDAKEKKKSAEDFEPRPSVTKYDA

## *Panax gingseng* ST2 (PgiST2). N-terminal, 2 repeats, 34 amino acids. Type not detected

MESRFALFALFSLLLIAGISDA

RLDPAGEYWQSIMKDEAMPEEIQGLIHDIRESVLPRTSTSTGHAHHNHKLVEENAKEKHFVN

DFEPRPNLSAYDDDLPTVSGSAYHDDASDKSFVNDFEPRPDATN

## *P. gingseng* ST3 (PgiST3). Full length, 2 repeats, 20 amino acids. Type III

MESCFALFALLALLLIASISDA

RHDPGEYWQSIMKDEEAMPEAIQGLVDIRESVLPRPNKKTDCHTSTSTGYAHHNDKLVEENAKEKHFVE

DFEPRPDVSAYHDDSDKSFVNDFEPRPDITNYHE

## *P. quinquefolius* ST2 (PquST2). Full length, 2 repeats, 20 amino acids. Type not detected

MESCFALFALLSLILIASISDA

RHDPGEYWQSIMKDEEAMPEAIQGLVDIRESVLPRPNKKTVCHTSTSTGYAHHNDKLVEENAKEKHFVE

DFEPRPNLSAYHDDSDKSFVNDFEPRPDISNYHE

## *Parthenium argentatum* ST1 (ParST1). N-terminal, 6 repeats, 23 amino acids. Type I

METSSSFIVFFSLTMVVFIIES

RPDSGIYWQNSIVQKSHSVTPLPWDKAHCQTSTETIIKGSSCDDASPYVNIKDNLVFDK

DFEPRPSATSYVDRTRLNGKKAFDAYFEPRPSATSYVTGVHGKKAFDT

EFEPRPSATSYVTGVNGKKTFDTDFEPRPSATSYVTGLDGKKVLNKDFEPRPSATSYVTGLNGKKTFDEDFEMRPSATSYVTGLSGK

## *Petunia axillaris* ST1 (PaxST1). Internal, 8 repeats, 26 amino acids. Type I

EKSFARDYDTMLKEETSFLT

YFEPRPTATAYYDDTGLKQERSSAKKFEQRPTVTAYNDDSGLKQKRSFATDFETRPTVAAYNDHTGLRQENSFVR

DFEPRSSATGYRNEDVGLKQKNSFFRDFEPRPSATGYSNEGAGLKQENSFVKDFEPRPSATGYRNEDVGLKQKNSFFKDFEPRPSATGYSNEGAGLKQEHSFVKDFEPRPSATGYRNEDVGL

## *Phaseolus vulgaris* ST1 (PvuST1). N-terminal, 6 repeats, 24 amino acids. Type I

MRPSLALLSLLFALLLTAITEA

RKDPGEYWKEIMKDQKMPEGLQGLLPFESENNAKTQEHLVKDSKHECEETEEKKVFTEDLDTKMGKPHFKSDFKND

KFEPRPSATKYDDSKVRLNV

DLDPRPSVTNYGDSKFRSNAIKNEDFEPRPSATKYGDFELKLSINKMD

DIEPRPSVTKYD

DFEPRPSATKYGDSEFGLNAIKND

DFEPRPSATKYGDX

## *Pisum sativum* ST1 (PsaST1). C-terminal, 8 repeats, 26 amino acids. Type I

MMSLNLDLVLLIDGGEGNKNMKLLVND

EFEPRPSVTKYDGDESYKNMKLSINDEFEPRPSATEYDGNEGYKNIKLPVNDEFEPRPSATKYDGDDGYKNMKLPINDEFEPRPSATKYDGDDGYKNMKLSVNDEFEPIPSVTKYDGDEGYKNLKLTINDEFEPRPSATKYDGDDGYQNMKLPINDEFEPRPSATKYDGDDGYKNMKLPLNDEFEPRPSATKYND

## *P. sativum* ST2 (PsaST2). Full length, 5 repeats, 26 amino acids. Subtype IIa

MSLRSAFALLPLFLFLIVANVES

RKENVGEYWKLVMKDQDMPEEIQGLLDASNIKNSKTHAKENMGAIG

EFEPRPYASAYGDNEIHAKENMGAIGEFEPRPNASAYGDNEIHANENKGATGEFEPRPNISAYGDNEIHANENKGAIGEFETRPNASAYGDNEIGAEFTD

DFEPRPSMTKYNA

## *P. sativum* ST3 (PsaST3). Full length, 3 repeats, 26 amino acids. Subtype IIa

MMSLRSAFALLPLFLFLIVANVES

RKDVGEYWKLVMKDQDMPEEIQGLLDASNIKNSKTHAKENKGAIG

EFEPRPNASAYGDNEIHANENKGAIGEFETRPNGSAYGDNEIGAEFTD

DFEPRPSMTKYNA

## *P. sativum* ST4 (PsaST4). Full length, 9 repeats, 26 amino acids. Subtype IIa

MMRLRPAFALLPLFLLLIITIVES

RKDLGKYWKLVMKDQDVSEEIQGLLDANIKKNFKTLRQSFDAKENKVVK

DFEPRPNVSVYGENDIDFMKNKAAIEEFEPKPNVSVYGNNNIDVEENNKGIEDFEPRPNVSTYGNNDIDNKKKDKEVEDFEPRPNISAYGNNDIDNKKKDKEVEDFEPRPNISAYGNNDIDNKEKEKAVEDFEPRPNVSAYGNNDINSRENEKVVEDFEPIPNVSAYGNNDIYNKEKKKVVEDFEPRPNVSAYGNNEIGAEFTE

DFEPRPSVTKYNA

## *P. sativum* ST5 (PsaST5). N-terminal, 3 repeats, 26 amino acids, Subtype IIa

MMRPALSLLPLFLLLIVGIVES

RKDLGEYWKLVMKQQDMPQEIQGLLNQNPKKEFQDSKAVFLM

MERRRKLSKILNKDLTFQL

MEKRTLMLRKRMGSLKILNQDLIYQLMEKMTSMLKKRKEPLKISNQYLTFQLMEKTTLMIKEKNEGIE

DFEPRPNISAYGENNIDVKEKKGVIEDFEPRPNISAYGENNIDVKEKNGTIEEFEPRPNISAYGENDIDVKEKKGAIEDFEPRPNISAYGENNFDDKXKEWGH

## *Populus deltoides* ST2 (PdeST2). Full length, 5 repeats, 25 amino acids. Type III

MKSSYTFFILFSLFSFANVIGA

RKDTGEYWRAVMKDQPMPEAIHGLIRETTLSSVSNEKADCHTTESNEKNNLVK

DFGPQPTGTSYDNGIKPAKDKSFSK

DFHPNTQFFLYNDGVVKGERSFAE

DVEPRPNVSVYHDDATLKGEKSFQEDFEPGPNLSVYDDGVGLKGKKLSSDDFEPRPSTTAYN

## *P. euphratica* ST2 (PeuST2). N-terminal, 4 repeats, 25 amino acids. Type III

MKSSFAFFVLFSLFSFSDVIGA

RKDTGEYWRAVMKDQPMPEAIQGLIRATTLSPVSNEKANCHTTESNEKHNFVK

DFRQQPTATSYDNGIEPAKNKYFSEDSDPKPNVSVYNDGVVKGERSFAE

DFEPRPNVSVFHDDATLKGEKSFTEDFEPRPSISVY

## *P. trichocarpa* ST2 (PtrST2). Full length, 5 repeats, 25 amino acids. Type III

MKSSFAFFVLFSLFSQFADVIGA

RKDTGEYWRAVMKDQPMPEAIQGLIRETTLSSVSNEKADCHTTESNEKNNFVK

DFGPQPTVTSYDNGIKPAKDKSFSKDFHPNTQLFLYNDGVVKGERSFAE

DLEPRPNVSVYHDDAALKGENSFPEDFEPGPNVSVYDDGVGLKGKKSFSDEFEPRPSVTAYSN

## *P. trichocarpa* ST3 (PtrST3). Full length, 7 repeats, 25 amino acids. Type III

MKSSYTFFILFSLFSFANVIGA

RKDTGEYWRAVMKDQPMPEAIHGLIRETTLSSVSNEKADCHTTESNEKNNFVK

DFGPQPTATSYDNGIKPAKDKSFSKDFHPNTQLFLYNDGVVKGERSFAK

DFGPQPTATSYDKGIKPAKDKSFSKHFHPNSQLFLYNDGDVKGERSFAE

DVEPRPNVSVYHDDATLKGEKSFQEDFEPGPNISVYDDGVGLKGKKSSSDDFEPGPRTTAAYNY

## *Prunus armeniaca* ST1 (PraST1). Full-length, 2 repeats, 26 amino acids. Type not determined

TRSLCAILALFSFLLFTKTIDS

RTDVGKYWKNVMKEQPMPQAIEGLLVDISDSTPKEKADCHEKVKKPFVEVEVEVEVE

EFEPRPSTTQYNDHETKAKLSSKYNA

GPKAKQSFAAKE

EFGPRPNPLIYSG

## *P. persica* ST2 (PpeST2). Full length, 7 repeats, 25 amino acids. Subtype IIb

MKSLCAILALFSLLLFVKTTYS

RRDVGKYWKNVMKEQPMPQAIEGLLVDISDSTPKEKADCHEKVKKPFVEVDVEVE

EFEPKPNALVYNAVAAKEDKQPFVKDFEPRPNALVYNAFAAKEDKQPFVKDIEPRPNALVYNAFAAKEDKQPFVKDFEPRPNALVYNAFAAKEDKQPFVKDFEPRPNALVYNAFAAKEDKQPFVKDFEPRPNAKFAAKEDQQPFVK

DFEPRPNALVYND

## *P. persica* ST3 (PpeST3). Full-length, 2 repeats, 25 amino acids. Type not determined

MKSLCAILALFSLLLFAKTTDS

RRDAGKYWKNVMQEQPMPRAIEGLLVDISDSTPKEKADCHEKVKKPFVEVE

EFEPRPSLTSYNHDETKAKLSSKDNAGPKAKQSFAAKEYKQPFEE

DFEPRPSASVYND

## *Q. petraea* ST1 (QpeST1). N-terminal, 7 repeats, 25 amino acids. Type I

MESRFAWLTLFLLLMFANTIES

RKDPGQYWTSVMKDQPMPKAIQGLVHLDSSPSKLSKNDNCHTSEGAIGKDQGEKPYVK

DFEPRPSVTVYVNDVGLETKNFVK

DFEPRPSVSAYNNDVGLKTQKKFVKDFEPRPSITAYVNDVGLETKNNFVKDFEPRPSLTAYVNDVGLETKNNFVKDFEPRPSVTAYINDVGLKNQNKFVKDFEPRPSVTTYVNDVGLEIKKKFVNDFEPRPSVT

## *Q. robur* ST1 (QroST1). Full length, 11 repeats, 25 amino acids. Type I

MTVDMESRFAWLTLFLLLLFANTIES

RKDPGEYWTSVMKDQPMPKAIQGLVHLDSSPSKLSKNDNCHTSEGAIGKDQGEKPYVK

DFEPRPSVTAYVNDVGLETKNFVK

DFEPRLSVTAYNNDVGLKTQKKFVKDFEPRPSVTAYVNDVGLETKNNFVKDFEPRPSVTAYINDVGLKNQNKFVKDFELRPSVTAYVNDVGLEIKKKFVNDFEPRPSVTAYVNDVGLETKKKFVKDFEPRPSVIAYINDVSPETNNFVK

DFEPRPSITAYVNDVGLETKKKFVNDFEPRPSVTAYVNVVDPQTKKKFVN

DFEQRPNISACNDDDVGSKDDKPFVEDFEPRPSASVYSE

## *Ribes nigrum* ST2 (RniST2). Full-lenght, 2 repeats, 26 amino acids. Subtype IIa

ASASSRCERRPSFFLILLSLFLMITTTIGA

RHDPGEYWRAVMKDQPMPEAIEGLINPNHHPQQHPLPLSSNPKTTSNCHTPTTTTTTNERNNENQLVVVIEKSKSPSVH

DFEPIPNLSAYKDEIRATEEKLVFDKDFEPIPNLSAYKDA

## *Ricinus communis* ST2 (RcoST2). N-terminal, 2 repeats, 25 & 26 amino acids. Type not determined

MNSSFPFIALFLFVLVTSTTDA

RKDVGEYWPRGVMKDQPLPEVAASSKINCHTTDDSELKNFVKEFKDPDETINHNDIKPGQSKSFFK

NFEPVPNLSVYNNDSILKEKKSFFVKDFEPRPDASVYDNDVGLKEDKSFAE

DFKARPNL

## *R. communis* ST3 (RcoST3). Internal, 2 repeats, 25 amino acids. Type not determined

MWESTGPRDVMKDQPLPETTACSRTNCHPTEDFELKNSVKYFKKTDGRIYHDNIKSVYDNNTTLKEEKTFTK

DYEPRPHISVYDNEIGLKEEKPFADDFEPRPDILIYSD

## *Robinia pseudoacacia* ST1 (RpsST1). Full length, 5 repeats, 25 amino acids. Type I

MKPVLAWLPFLFLFLFAATIES

RKDQGEYWKTIMKDEEMPEAIQGLLQLNSKHEG

EEEPLVTNTRYVIEKEVFTE

EFEPRPNLSAYDDDHVDTKENKKYKDIEPRPSVTSYREQIMK

IFEPRPSVTAYNGDGYKSEKPQVNSEFEPRPSASKYND

## *Rosa hybrida* ST2 (RhiST2). N-terminal, 4 repeats, 16 amino acids. Type III

MNSLCAILASLSLLLFTTTVES

RIGAGGYMKNSIEKQPTPGDKTEKANCNENGKPNIEFEIV

EFEPRPDATIYHGDIVEFEPRPDATIYHGDIVEFEPRPDATIYHGDSEVTESRTLKDELESKAIGNMHPKDNGPKDKLPFEVKANRHAFEE

DFEPRPNVSVYKD

## *Salvia miltiorrhiza* ST2 (SmiST2). Internal, 5 repeats, 25 amino acids. Subtype IIa

PFVR

EFEDRPSVNVKLTHEKSYSK

DFEPRPNVSAYTNDVKRTDEKSYTKDFEPRPNVSAYTDDVKVTDEKSYTKDFEPRPNVSAYTDDVKLTDEKSYTKDFEPRPNVSAYTDDVNSKDEKSYTKDFEPRPNVSAYTDDVNSKDSIYTIGAKAEK

## *Saussurea medusa* ST2 (SmeST2). Internal, 4 repeats, 26 amino acids. Subtype IIa

TPKAPKSLILVEYQKMESPLCFLALFSIVMIVVSVDARPNPVEFMQDSSVRRGTSTPPLPTKKSDCRTLAKTTNHRKSFNE

EFEPRPNISAYDNEVNQDANNK

DFEPRPNISVYDDSISLKGKKMSDE

EFEPRPNISVYDNSVGLKISKRTFEEEFEPRPNVSVYEG

## *Senecio aethnensis* ST1 (SaeST1). Full length, 7 repeats, 25 amino acids. Type I

MKYSTLSFLALFSLIMITIIVES

RPDNGEYWQDSLVRGGTSASPLPTKKTHCHTLAEAKNHIKSFNK

DFEPRPSATGYDNNVSLKDERRFDKDFEPRPSASGYDNERGLNGKKSFDENFEPRPSAGGNDNGKGLKGYRTFDKAFEPRPNLSGYENGIGLKGDKTFDKDFEPRPNLSGYDNGMGVKGEITFGKDFEPRPSASGYDDKASLENKSSVDKEFKAIASVTGYVV

## *S. chrysanthemifolius* ST1 (SchST1). N-terminal, 4 repeats, 25 amino acids. Type I

MKYSTLSSLALFSLIMIAIIVEA

RPDNGEYWQDSLVQRGTSVSSLTTKKTHCYTLANAKNHIKSFNE

DFEPRPSATGYDNGIGLKDERMFDKDFEPRPSATGYDNEAGLKGERMFNKYFEPRPSATGYDNGMGIKGEKTFDKEFEPRPSATGYDNGMSL

## *Sesamum inducum* ST2 (SinST2). N-terminal, 4 repeats, 25 amino acids. Type not determined

MRSTVFILAGLFALFANTINA

RKDPGAYWEAVMKDEPMPQVIQGLVDDASKVTSPLSPQKNHCNTTPTNSR

TFQPTPNLSSYNDDDKLKAQRSFRNDFEPRPSASAYTDASEVKQEKSFVKDFEPRPNLTAYTDKSDAEKDK

DFEPRPTSSAYNDNKDM

## *Solanum lycopersicum* ST1 (SliST1). Full length, 6 repeats, 26 amino acids. Type I

MKLPVALILLFSLALYASSTDA

RKDPXEYWRAVMKDEPMPEAIKHLMPRHSVPLSKEKTDCYTSSSVGGE

ASEPKPNLYVYHNDANLKGAEKSLFTR

DFEPRPSATGYHDDDVGLKEKSLFTRDFEPRPSLTGYHDDDVGLKEKSLFTRDFEPRPSVTGYHDDDVGLKEKSLFTRDFEPRPSVTGYHDDDVGLKQEKSFAEXFEXXPNLSVYHD

## *S. melongena* ST1 (SomST1). N-terminal, 3 repeats, 22 amino acids. Type I

MKLIIAFILLFSLALYTDA

RKNPGEYWRDVMKDEPMPKAIQHLMPQSHKEKSDCHKS

SFEPIPNVSSYHGDDIDLKQEKDFEPRPSTTGYHDDDVGLKLEKDFEPRPSTTGYHDDDIGLKQEKDLNHDQVLLAIIMTTLVSNKKKILNQNQILLVIMMMTMLLS

## *S. melongena* ST2 (SomST2). Full length, 5 repeats, 22 amino acids. Subtype IIa

MKLFIALILLFSLALYTDA

RKDPGEYWRDMMKDEPMPKAIQHLMPQSHKEKSDCHKS

SFEPIPNVSSYHGDEAGLKQEKTFEPRPNVSSYHGDDAGLKQEKTFEPRPNISSYHDNDVGLKQKKDFEPRPNVSSYHGDDIGLKQEKNFKPRPNISSYHE

## *S. nigrum* ST2 (SniST2). Internal, 5 repeats, 22 amino acids. Subtype IIa

DA

RKDLGEYWRDVMKDEPMPKAIQHLMPQSHKEIKTDCHKS

SFEPIPNVSSYHGDDVVLKQEKDFEPRSNVSSYHDDDVGLKQEKDFEPRPNVPSYHGDDANLKQEKDFEPRPNVSGYHSDDVGLKQEKDFEPRPNVSSYHGNDADLK

## *S. tuberosum* ST2 (StuST2). Full length, 9 repeats, 24 amino acids. Subtype IIa

MKSHIALILLFSLALYTDA

RKDPGEYWRDVMKDEPMPKAIQHLMPQPHKEKIDCHKS

SFEPIPNVYSYHNEEDGLKQEKDFELRPNVSGYHDDDVGLQQEKVFEPRPNVSGYHDDDVGLKQEK

DFEPRPNVSSYHDDDNVGLKQEKDFEPRPNVSSYRDDDNVGLKQEKDFKPRPNVSSYHDDDHVGLKSKKDFEPRPNVSKYHDDDNVGLKQEKDFEPGPNVSSYPENDNVDLKQEKEFQAKPNASWYPN

## *S. tuberosum* ST3 (StuST3). Full length, 5 repeats, 22 amino acids. Type III

MKSLFALILLISLAPHASSTDA

RRDPGEYWNIVMKNEPMPIAIKHLMPRYDV

PFEPRPTVTAYYHDDASLKQEKSFEPRPTATSYHDNEVSLKGEK

SFEPRPSATTYHHDDVGLKQEKSSFTK

DFEPRPTATSYRDDEAGLKGEKSFEPRPNVSMYND

## *Striga asiatica* ST2 (SasST2). Internal fragment, 6 repeats, 25 amino acids. Subtype IIa

SFVK

DFEPRPNISAYTDEAKLGDDKSFDKDFEPRPNVSAYTDDVKTRQEKSFSKDFEPRPSITAYTDDAKEENKKSFVKDFEPRPNISAYTNEAKLGDDKSFDKDFEPRPNVSAYTDEVKSDDKKTFVKDFEPRPNVSAYTDDVKTEDEKSFGSAVEKVIDVIGRI

## *S. hermonthica* ST2 (SheST2). Internal fragment, 6 repeats, 25 amino acids. Subtype IIa

KLGDEKSFDK

DFEPRPNVSAYTDDAKSRQGKSFVKDFEPRPNISAYTDDDKEENKKSFVKDFEPRPNVSAYTDEAKLGDDKSFDKDFEPRPNVSAYTDEVKSDDKKTFVKDFESRPNVWAYTNDVKTESGKSFRKDFEPRPNVSAYTNDVKTDDEKSFGSAVEKVIDVIGRI

## *Taraxacum kok-saghyz* ST1 (TkoST1). C-terminal, 8 repeats, 25 amino acids. Type I

HTLKEAGYQRKSYNQ

DLKPLSSVSVYDNNVGLEYKKTFAEDFKRRPSVTGYINNAGLKDKRSFDEQFEPRPSVTGYDNGADLKDKMTFDEEFEPRPSATGYDNNASLKNKRTFDEEFEPRPSVTGYDNNVGLKGKMTFDEEFEPRPSVTGYDNGADLKYKRTFNEEFEPRTSVTGYDSKADLKNKRTFDEEFEPRPNVTAYKG

## *T. oficinale* ST1 (TofST1). Full length, 6 repeats, 25 amino acids. Type I

MESHLSFLVLFSLVMITVNVYA

RPNPQKYWQDSNVRGALLALPHPTKRSHDCNTLKEVGYQRKSYNQ

DVKPLSSVSVYDNNEGLKDKKTFAE

DFKQRPSVTSYNNNAGLKDKRSFDEQFEPRASVTGYDNGVDLKDKMTFDEGFQPRPSVTGYDNGVGLKIKSTFNEEFEPRPSVTGYDSNADLKNKRTFDEEFEPRPNVTAYKG

## *T. oficinale* ST2 (TofST2). N-terminal, 5 repeats, 25 amino acids. Subtype IIa

MESHKSFLAIFSLVMITVTVIVNA

RPNPEEYWQDSHVRGGSLVSPHPTKRSHCNTLAEARNERKSSNK

DFEPRPNISVYDNDVGLKRKKTIDNSAGLKDKKTFAE

DFEPRPNISVYENGESLKGRKAYDEDFEPRPNASVYENGESLKGKNTFVEDFEPRPNISVYENGEGLKDKKTLDEDFEPRPNISVYDNNAGL

## *Theobroma cacao* ST2 (TcaST2). Full length, 4 repeats, varied amino acids. Subtype IIa

MKSFLSFFAFLSLLLFANTIAAA

RKDAGEYWRAVMREQAMPEAIEALVRIDAATSSKEKTDCHTPTSFELKEEKILVE

AFEPRPNEGEKKSFAD

YFEPRPNVSAYGDDADLKAEKSSS

FTKDRPSIPAYGDDAGLKGEKKSFVS

DFEPGPNITVYHD

## *Trifolium pratense* ST2 (TprST2). N-terminal, 5 repeats, 26 amino acids. Subtype IIa

MRHAFALLPLLLFLFAASIES

RKDLGEYWKLIMKDQDMLEEIQGLLIANTKKILKPVVKDTQANLEVNVFRE

DLEPRPNISAYEDDTKENKKDFK

DFEPRPNISAYGNNDIDVKEKKKIVKDFEPRPNISAYGDNDIDVKENNGTTKDFEPRPNISAYGENDIDAKEKKEATKDFKPRPNISAYG

## *T. pratense* ST3 (TprST3). N-terminal, 7 repeats, 24 + 16 amino acids. Subtype IIb

MRPALALLPLLLLLFAATVES

RKDLGEYLKLAVKDQHTAEYIQGLLNFNDKKNLKTQKPNINVFGDKEIDAKENKEVIQ

DFESRPNGFVYHGIDAEEKKGAIKDFEPRPNAFSYKGAIK

DFEPRPNVFHYHGIDAEEKKGAIKDFEPRPNAFSYKGAIK

DFEPRPNAFHYHGIDAEEKKGAIKDFEPRPNAFSYKGAIK

DFEPRPNVFHYHGIDAEE

## *Triphysaria versicolor* ST2 (TveST2). Internal, 10 repeats, 25 amino acids. Subtype IIa

KRPSSN

DFEPRPSVTAYVDNLKSKGGNSYKNDFEPRPSVTAYVDNLKSKGENSYKNDFEPRPSVTAYVDNLKSKGENSYKN

DFKPIPNVSAYTDDVKKNVKKNDKFFGK

DFEPIPNISAYTDDVKKDDKIFGK

NFEPIPNVSAYTDDVKKEDDKFFGKNFEPIPNVSAYTDDMKKEDDKSFDKNFESIPNISAYTDDVKKYDKSFGK

EFEPRPNISPYNDDVKKQDVKSVGKDFYPRPNISAYTDDVKSDGK

## *Vigna unguiculata* ST1 (VunST1). Full length, 12 repeats, 12 amino acids. Type I

MRSSLAMFSLLFAFLLSGTIEA

RKDPGEYWKEIMKDQQMPEGLQGLLPFQSENNPKTQEQLVKDSKHECEEPLATNVGELKSSAKKNE

DIEPRPSVTKYD

DFEPRPSVTKYGKSEFKSSATKKD

DIEPRPSVTKYGDFELKLIASKKD

DIEPIPSVTKYG

DFEPRSSLMMYDDFGVKLSANKNDDIEPRPSVTKYS

DFEPRSSITKYDDFEFKSSASKKNDIEPRPSLTKYD

DFEPRPNISKYG

DFEPRPSITKYN

DFEPRPSITKYDDFESKPSTTKVDDFEPRPNISKYDD

## *V. unguiculata* ST2 (VunST2). N-terminal, 12 repeats, 26 amino acids. Subtype IIa

MRYFLTLLPLLFLFLFVTNVES

RNMGGEYWKMAAKNQDIAEEFKGLPTHENDEIYVKRERKDVK

ESDLNSNVATYFNKGIDIESKKKVTR

TFEPRPNVSAYSEKDIDCMEKKKMKKHFEPRPNVSAYSDNEVGVKDKNKVMKHFEPKLNASAYGDNEVDVKEKNKSVKDFEPRPNVSAYGDNEIGVQEKKKTVKDFEPRPNVSAYGDNDIDAKKKKEVKEDFEQRPNVSAYGDNDIDAKKKKEVKEDFKPRPNVSAYGDNNIDAKKKKKVKEDFEPRPNVSAYSDNDIDAKKKKEVQEDFEQRPNVSAYGDNDIDAKKKKEVKEDFKPRPNVSAYGDNNIDAKKKKKVKEDFEPRPNVSAYSDNDIDAKKKK

## *Vitis amurensis* ST2 (VamST2). Full length, 3 repeats, 25 amino acids. Subtype IIa

MESSFALFVILLLVLVGNTIAA

RKDPGEYWKDAMKDQPMPKAIEGVLSAKDPNCHTATEASNEQADQLLRDFEQKVEKAFAE

DFEPRPNVSVYHDDSKVGEEKSFVK

DFEPGPNLSVYHDDEVASKGDKSFVNDFEPRPNLSVYNH

## *V. vinifera* ST2 (VviST2). Full length, 3 repeats, 25 amino acids. Subtype IIa

MESSFALFVILSLVLVGNTIAA

RKDPGEYWKDAMKDQPMPKAIEGVLSAKNPNCHTATEASNEQADQLLKDFEQKVEKAFAE

DFEPRPNVSAYHDDSKVGEEKSFVK

DFEPGPNLSVYHDDEVASKGDKSFVNDFEPRPNLSVYNH

## *V. vinifera* ST3 (VviST3). Full length, 3 repeats, 25 amino acids. Subtype IIa

MESSFALFVILSFVLVGNTIAA

RKDPGEYWKDAMKDQPMPKAIEGVLSAKNPNCHTATEASNEQADQLLKDFEQKVEKAFAE

DFEPRPNVSVYHDDSKVGEEKSFVK

DFEPGPNVSVYHDDEVASKGDKSFVNDFEPRPNVSVYND

## *V. vinifera* ST4 (VviST4). Full length, 2 repeats, 25 amino acids. Subtype IIa

MAYSLAFFTIFSLLLVGNAIGG

RKEPGEYWRDVMKDEPMPKAIQGLLPEDQSSSPSSKKLDCQTTTEARNGDDVVKGFEPKKEKVFWVYEDEDAKLTEEKSFVK

DLEPRTNISAYHDKGATKEEKPFVGDYEPRPNISAYNE

## *V. vinifera* ST5 (VviST5). Full length, 2 repeats, 25 amino acids. Subtype IIa

MASSLAFFTIFPLLLVGNAIGG

RKEPGEYWRDVMKDEPMPKAIQGLLPEDQSSSLSSKKTNCQTTTEARNGDDVVKGFEPKKEKVFWVYDDEDAKLTEEKSFVK

DLEPWTNISAYHDKGATKEEKPFVGDYEPRPNISAYNE

## *V. vinifera* ST6 (VviST6). Full length, 2 repeats, 27 amino acids. Subtype IIa

MASSLAFFTIFSLLLVGNAIGG

RKEPGVYWRDVMKDEPMPKAIQGLLPEDQSSSLLSKKPNCQTIPGATNGGDIVKGFEPKKEKVFWVYDDEDAKLTEEKSFVK

DLEPKTNVSAYRDKVTTKEEKEKPFVGDFEPRPNVSAYNE
